# Computational-experimental integration identifies potent carbohydrate-hydrolyzing enzyme inhibitors from *Nardostachys jatamansi*: molecular docking, dynamics and pharmacokinetic predictions

**DOI:** 10.3389/fphar.2025.1713452

**Published:** 2026-01-12

**Authors:** Muhammad Javid Iqbal, Iqra Malik, Gonzalo Bernal, Alexis M. Salas-Burgos, Luis A. Salazar

**Affiliations:** 1 Doctoral Program in Sciences, Specialization in Applied Cellular and Molecular Biology, Universidad de la Frontera, Temuco, Chile; 2 Department of Basic Sciences, Center of Molecular Biology and Pharmacogenetics, Faculty of Medicine, Universidad de la Frontera, Temuco, Chile; 3 Department of Pharmaceutical Chemistry, Faculty of Pharmacy, Bahauddin Zakariya University, Multan, Pakistan; 4 Oncology Progression Laboratory, Pharmacology Department, Biological Science Faculty, Concepcion University, Concepcion, Chile; 5 Centro Territorial de Investigación Oncológica Integrativa (CTIOi), Santiago, Chile

**Keywords:** molecular docking, density functional theory, enzyme inhibition, natural products, molecular dynamics simulation, ADMET profiling, antidiabetic agents, *Nardostachys jatamansi*

## Abstract

**Background:**

Current α-glucosidase and α-amylase inhibitors demonstrate limited therapeutic efficacy and significant gastrointestinal side effects, necessitating identification of novel antidiabetic agents. This study employed integrated computational and experimental approaches to evaluate carbohydrate hydrolyzing enzyme inhibitory potential of *Nardostachys jatamansi* and its phytochemicals.

**Material and methods:**

Plant extracts were evaluated through enzymatic assays against α-glucosidase and α-amylase. Virtual screening of 144 phytochemicals employed molecular docking, followed by molecular dynamics simulations (100 ns) and density functional theory calculations at B3LYP/6-311++G(d,p) level. ADMET profiling assessed drug-likeness potential.

**Results:**

*N. jatamansi* extract demonstrated superior enzyme inhibition compared to acarbose: IC_50_ values of α-glucosidase 61.7 ± 3.9 μg/mL and α-amylase 81.3 ± 4.7 μg/mL versus 132.6 ± 7.8 μg/mL and 112.1 ± 6.2 μg/mL respectively. Molecular docking identified Virolin with selective α-glucosidase affinity (−9.6 kcal/mol) and Nardostachysin showing high α-amylase binding (−9.5 kcal/mol). Molecular dynamics revealed Nardostachysin-α-amylase complex stability (ΔG = −158.51 kcal/mol) throughout simulation, while Virolin-α-glucosidase complex showed late-stage dissociation. DFT calculations revealed HOMO-LUMO gaps of 4.78 eV (Virolin) and 4.57 eV (Nardostachysin) with distinct dipole moments of 4.83 and 6.29 Debye respectively. ADMET analysis confirmed favorable drug-likeness with complete Lipinski compliance and zero PAINS alerts for both lead compounds.

**Conclusion:**

*N. jatamansi* extract demonstrated experimentally superior enzyme inhibition compared to acarbose, while computational analysis identified Virolin and Nardostachysin as promising drug candidates, establishing a validated integrative approach for accelerating natural product antidiabetic lead discovery.

## 1 Introduction

Diabetes mellitus affects 537 million adults globally and continues to escalate despite therapeutic advances, with type 2 diabetes accounting for approximately 90% of cases (Hossain et al., 2024). The pathophysiology centers on impaired glucose homeostasis, where postprandial hyperglycemia serves as both a diagnostic marker and a primary contributor to diabetic complications (Banday et al., 2020). Two key enzymes regulate postprandial glucose levels: pancreatic α-amylase initiates starch hydrolysis into oligosaccharides, while intestinal α-glucosidase completes the conversion to absorbable glucose (Mittal et al., 2025). Inhibiting these enzymes delays carbohydrate digestion, reduces glucose absorption rate, and consequently attenuates postprandial glycemic spikes (Tolmie et al., 2021).

The strategic targeting of these carbohydrate hydrolyzing enzymes offers distinct advantages in diabetes prevention and early intervention (Tundis et al., 2016). Unlike insulin secretagogues or sensitizers that require functional β-cell mass, enzyme inhibitors provide a mechanism-based approach suitable for prediabetic states and early-stage type 2 diabetes (Park et al., 2013). By modulating carbohydrate digestion at the intestinal level, these inhibitors prevent the postprandial glucose excursions that drive progressive β-cell dysfunction and insulin resistance key pathophysiological processes in diabetes progression (Dalle et al., 2023). Furthermore, this peripheral mechanism minimizes systemic metabolic perturbations and offers a preventive strategy by addressing the glycemic burden before pancreatic exhaustion occurs. Among numerous diabetes-related protein targets (including DPP-4, SGLT2, glucagon receptors, and GLP-1 pathways), carbohydrate-hydrolyzing enzymes represent first-line defense targets that directly intercept dietary glucose absorption, making them particularly relevant for prevention-focused interventions (Zhang et al., 2025).

Current α-glucosidase inhibitors including acarbose, miglitol, and voglibose effectively reduce postprandial glucose but present significant clinical limitations (Das et al., 2018). Gastrointestinal adverse effects occur in 50%–70% of patients, primarily due to undigested carbohydrate fermentation in the colon (Yap and Gan, 2020). Additionally, these drugs show modest HbA1c reductions (0.5%–0.8%), potential hepatotoxicity with prolonged use, and considerable cost barriers in developing nations. These constraints necessitate identifying alternative therapeutic agents with improved safety and comparable or superior efficacy (Miller and Joubert, 2022).

Natural products represent a validated source for antidiabetic drug discovery, with approximately 800 plants demonstrating glucose-lowering properties in ethnopharmacological surveys (Nazar et al., 2024). Plant-derived compounds offer structural diversity, multiple mechanisms of action, and generally favorable safety profiles developed through evolutionary selection (Nasim et al., 2022). The success of metformin, derived from *Galega officinalis*, exemplifies the potential of plant-based antidiabetic agents (Hajjar et al., 2013). Recent advances in analytical techniques and computational biology have accelerated the identification and characterization of bioactive compounds from medicinal plants (Zhang et al., 2024).

**FIGURE 1:**
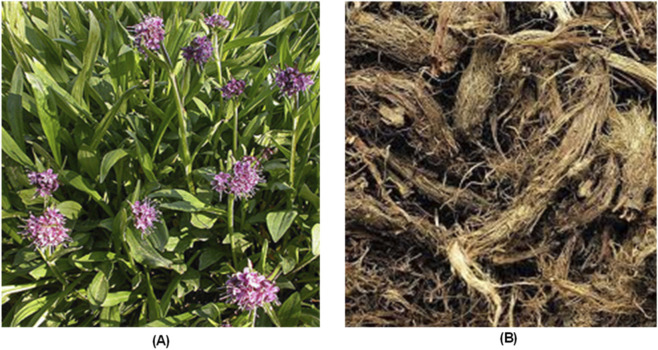
*N. jatamansi* rhizomes and whole plant from Himalayan region **(A)** growing plant **(B)** dried whole plant.

*Nardostachys jatamansi* DC. (Caprifoliaceae), a perennial herb endemic to the Himalayas (3000–5200 m altitude), has been extensively utilized in Ayurvedic, Unani, and Tibetan medicine systems (Dhiman and Bhattacharya, 2020). The plant, known as *jatamansi* or spikenard (Figure 1), grows in alpine regions of Pakistan, India, Nepal, Bhutan, and Tibet, where its rhizomes are harvested for medicinal purposes (Chauhan et al., 2021). Historical documentation extends from Vedic texts (1000–800 BCE) through classical medical treatises, indicating its use for neurological, cardiovascular, and metabolic disorders (Shah, 2023).

Phytochemical investigations have identified diverse bioactive constituents in *N. jatamansi* rhizomes, predominantly sesquiterpenoids including jatamansone, nardostachone, aristolone, and the kanshone series (Yoon et al., 2018a). Additional compounds include iridoid glycosides, phenolic acids (chlorogenic acid), and novel nardosinone type sesquiterpenoids with documented biological activities (Li et al., 2021; Yoon et al., 2018b). These compounds exhibit synergistic pharmacological effects through multiple molecular targets (Gottumukkala et al., 2011).

Recent studies demonstrate significant antidiabetic properties of *N. jatamansi*. In db/db mice models, the extract reduced blood glucose, glycosylated hemoglobin, and improved insulin sensitivity through AMPK activation and hepatic gluconeogenesis suppression via PEPCK and G6Pase downregulation (You et al., 2018). The plant protects pancreatic β-cells from streptozotocin and cytokine-induced damage through NF-κB pathway inhibition, preserving insulin secretion capacity (Song et al., 2010). Additionally, *N. jatamansi* exhibits potent antioxidant activity, reducing oxidative stress markers associated with diabetic complications (Sharma and Singh, 2012; Razack et al., 2015). The plant demonstrates complementary activities relevant to diabetes management, including anti-inflammatory effects through 5-lipoxygenase inhibition (Shin et al., 2015), neuroprotection against diabetic neuropathy (Anupama et al., 2022), and cardiovascular protection (Singh et al., 2020). Recent investigations revealed antihypertensive properties through PDK1-Akt-eNOS signaling, addressing diabetes-associated vascular complications (Fang et al., 2022).

While α-amylase inhibitory activity has been reported for *N. jatamansi* extracts (Bose et al., 2019), comprehensive characterization of enzyme inhibitor interactions remains absent. Critically, no studies have investigated α-glucosidase inhibition by *N. jatamansi*, despite this enzyme’s central role in postprandial glucose control. The specific bioactive compounds responsible for enzyme inhibition, their binding mechanisms, structure-activity relationships, and pharmacokinetic properties remain uncharacterized. These knowledge gaps limit the development of *N. jatamansi* derived antidiabetic agents.

This study employs an integrated approach to evaluate *N. jatamansi* extract against α-amylase and α-glucosidase through *in vitro* enzyme assays, molecular docking to identify protein-ligand interactions, molecular dynamics simulations for binding stability assessment, DFT for selective compounds reactivity and ADMET profiling for drug-likeness evaluation. This comprehensive investigation aims to identify bioactive constituents, elucidate their inhibitory mechanisms, and assess their potential as antidiabetic drug candidates.

## 2 Materials and methods

### 2.1 Chemical reagents

The following chemical reagents were used for the *in vitro* enzymatic assays: α-Glucosidase from *Saccharomyces cerevisiae* (Cat. No. G5003), α-amylase from porcine pancreas (Cat. No. A3176), and the standard inhibitor acarbose (Cat. No. A8980) were purchased from Sigma-Aldrich (St. Louis, MO, United States). p-Nitrophenyl-α-D-glucopyranoside (pNPG), soluble starch, sodium carbonate (Na_2_CO_3_), 3,5-dinitrosalicylic acid (DNS) reagent, and dimethyl sulfoxide (DMSO) were also obtained from Sigma-Aldrich. Phosphate buffers (0.1 M, pH 6.9) were prepared using analytical-grade chemicals. Spectrophotometric measurements were performed using a BioTek Synergy H1 microplate reader (Agilent, Santa Clara, CA, United States).

### 2.2 Sample collection

Whole plants of *N. jatamansi* (roots, rhizomes, stems, and leaves) were collected in the Murree Hills, Punjab Province, Pakistan, in September 2024. Identification was based on morphology using regional floras (Flora of Pakistan; Flora of India) and cross-checked against the NCBI Taxonomy database (Taxon ID: 179860). The determination was confirmed by botanists at the Department of Botany, University of Agriculture, Dera Ismail Khan (Khyber Pakhtunkhwa, Pakistan). A voucher specimen (No. NJ-045/2024) has been deposited in the Departmental Herbarium of the same institution.

### 2.3 Extract preparation of *N. jatamansi*

Whole plants of *Nardostachys jatamansi* including roots, rhizomes, stems, and leaves were used to ensure a comprehensive phytochemical profile. The cleaned plant material was dried in a hot-air oven at 50 °C until a constant weight was achieved (typically 48–72 h). This moderate drying temperature was deliberately chosen to remove moisture effectively while minimizing the degradation of heat-labile and volatile compounds. A 90% methanolic hydroalcoholic solvent was employed for extraction due to its high efficiency in recovering a broad spectrum of medium- to low-polarity secondary metabolites, including the targeted sesquiterpenoids. Exhaustive extraction was performed using a Soxhlet apparatus, which allows continuous solvent recycling and ensures maximum yield. For each extraction, 10 g of the powdered plant material was extracted with 150 mL of 90% methanol in a thimble for 24 h under reflux. The resulting dark-green extract was concentrated under reduced pressure using a rotary evaporator at 50 °C following a modified procedure by Kadan et al. (2016), until complete solvent evaporation. The dried extract was stored at 4 °C for further experiments. A stock solution was prepared in 90% methanol, and the percentage yield was calculated according to Equation 1.

$$\text{Yield }\left(\%\;w/w\right)=\frac{\text{Weight}\;\text{of}\;\text{dried}\;\text{extract}\;\left(g\right)}{\text{Weight}\;\text{of}\;\text{initial}\;\text{plant}\;\text{material}\;\left(g\right)}\times 100$$ (1)

### 2.4 α-glucosidase inhibition activity

The inhibitory activity against α-glucosidase was evaluated spectrophotometrically, following an established protocol adapted from Kim et al. (2004). Briefly, test samples (plant extracts) and the reference inhibitor (acarbose) were prepared at varying concentrations ranging from 7.82 to 500 μg/mL in 0.1 M sodium phosphate buffer (pH 6.9). A 100 µL aliquot of each sample solution was combined with 15 µL of α-glucosidase solution (0.1 U/mL, pre-incubated at 37 °C for 10 min) in a microplate well. The enzymatic reaction was initiated by adding 250 µL of 20 mM p-nitrophenyl-α-D-glucopyranoside (pNPG) substrate. After incubating the reaction mixture at 37 °C for exactly 10 min, the reaction was terminated by adding 100 µL of 0.1 M sodium carbonate (Na_2_CO_3_). The amount of p-nitrophenol liberated by enzymatic hydrolysis was quantified by measuring the absorbance at 405 nm using a microplate reader. The percentage inhibition of α-glucosidase activity was calculated using the following formula in Equation 2.

$$\%\;\alpha -\text{glucosidase inhibition}=\frac{\left[{\text{ABS}}_{\text{blank}}{\;-\;\text{ABS}}_{\text{test}}\right]\;}{\left[{\text{ABS}}_{\text{blank}}\right]\;}\times 100$$ (2)

### 2.5 α-amylase inhibition activity

The inhibitory activity of plant extract against α-amylase was assessed following Adisakwattana et al. (2012), with minor modifications. Serial dilutions of both *N. jatamansi* extract and acarbose were prepared in 0.1 M sodium phosphate buffer (pH 6.9) to achieve final concentrations of 7.82, 15.63, 31.25, 62.5, 125, 250, and 500 μg/mL. In 96-well microplates, 50 μL of each test concentration was mixed with 50 μL of α-amylase solution (1 U/mL in phosphate buffer) and pre-incubated at 37 °C for 10 min. The reaction was initiated by adding 100 μL of 0.5% soluble starch solution, followed by incubation at 37 °C for 15 min. The reaction was terminated by adding 50 μL of 3,5-dinitrosalicylic acid (DNS) reagent and boiling for 5 min. After cooling, absorbance was measured at 540 nm using a microplate reader. Controls included: (1) 100% enzyme activity (buffer replacing inhibitor), (2) blank (buffer replacing enzyme), and (3) background (buffer replacing both enzyme and substrate). Percentage inhibition was calculated using formula given in Equation 3.

$$\%\;\alpha -\text{amylase inhibition}=\frac{\left[{\text{ABS}}_{\text{blank}}{\;-\;\text{ABS}}_{\text{test}}\right]\;}{\left[{\text{ABS}}_{\text{blank}}\right]\;}\times 100$$ (3)

### 2.6 Statistical analysis

All enzyme inhibition assays were performed in technical triplicate within each independent experiment. Data represents mean values ± standard deviation (SD) from three independent biological experiments (*n* = 3). Raw data calculations and initial statistical analyses were performed using Microsoft Excel 2016 (Microsoft Corp., United States).

IC_50_ values were determined using four-parameter logistic regression (dose-response curves) in GraphPad Prism 8.0 (GraphPad Software, San Diego, CA). Statistical comparisons between *N. jatamansi* extract and acarbose at individual concentrations were conducted using unpaired Student’s t-test. Statistical significance was defined as *p ≤ 0.05, **p ≤ 0.01, and ***p ≤ 0.001.

### 2.7 Computational studies

Molecular docking studies were performed using the AutoDock Vina engine integrated into PyRx 0.8 to investigate the binding interactions of phytochemicals with the key carbohydrate-hydrolyzing enzymes α-amylase and α-glucosidase, which are crucial therapeutic targets for diabetes management (Sargis Dallakyan and Olson, 2015). Catalytic binding pockets of both target proteins were identified using BIOVIA Discovery Studio v24.1.0.23298 based on PDB site records and known active sites, and the docking grids were centered on the biologically relevant catalytic clefts where the reference inhibitor acarbose binds. (Shaweta et al., 2021). For α-amylase (PDB ID: 1B2Y), the grid was centered at (x = −37.0, y = −32.0, z = −31.0) and encompassed the catalytic triad Asp197, Glu233, and Asp300 (Rydberg et al., 2002), whereas for α-glucosidase (PDB ID: 2ZE0), the grid was centered at (x = −3.0, y = 18.0, z = −17.0) targeting the conserved catalytic site containing Asp199, Glu256, and Asp326 (Nguyen et al., 2022; SULONG et al., 2025). In both cases, the grid box dimensions were set to 20 Å × 20 Å × 20 Å to ensure complete coverage of the inhibitor-binding region. All phytochemicals were energy minimized within PyRx using the Open Babel tool with the Universal Force Field (UFF) and Conjugate Gradients algorithm, converted to .pdbqt format and assigned Gasteiger partial charges. The receptor proteins were treated as rigid while the ligands were flexible, allowing conformational sampling during docking. The exhaustiveness parameter was set to 8, and nine docking poses were generated for each ligand, with the pose showing the lowest binding energy selected for detailed interaction analysis. Protein–ligand complexes were visualized and analyzed in BIOVIA Discovery Studio to identify key interactions and binding residues, and conformations with the most favorable docking scores and RMSD values were considered potential lead candidates for further molecular dynamics simulations and *in vitro* validation.

#### 2.7.1 Collection and optimization of bioactive compounds

For the current study, a total of 144 biologically important active phytochemicals were collected from literature and different databases (detailed in Supplementary File S1). The IMPPAT (Indian medicinal plants, phytochemistry and therapeutics) database (Bose et al., 2019) (https://cb.imsc.res.in/imppat/; accessed on 10 January 2025), and TCMSP (the traditional Chinese medicine systems pharmacology) database (Ru et al., 2014) (https://tcmsp-e.com/tcmsp.php; accessed on 15 January 2025) were used to search the active phytochemicals with reported antidiabetic activity. PubChem database (Kan et al., 2023) (https://pubchem.ncbi.nlm.nih.gov/; accessed on 01 February 2025) was used to retrieve the chemical structures of these plant phytochemicals in.sdf format. The phytochemicals’ energy was minimized before the molecular docking study.

#### 2.7.2 Retrieval and preparation of receptor proteins

α-glucosidase and α -amylase were selected as receptor proteins and used for molecular docking studies. The three-dimensional (3D) structures of receptor proteins (i.e., α-glucosidase with PDB ID: 2ZE0 and α-amylase with PDB ID: 1B2Y) were downloaded from Protein Data Bank in.pdb format (https://www.rcsb.org/; accessed on 03 March 2025) (Rose et al., 2021). The bacterial α-glucosidase structure (2ZE0) was utilized due to its high-resolution crystallography (1.60 Å), conserved GH13 catalytic machinery shared with human maltase-glucoamylase (Nguyen et al., 2022; SULONG et al., 2025), and extensive validation as a computational screening target for α-glucosidase inhibitor discovery. The binding pockets of the receptor proteins were predicted using BIOVIA Discovery Studio (Shaweta et al., 2021) To further prepare the receptor proteins for molecular docking, already bound ligand(s) removed (if any), the water molecules were deleted, hydrogen atoms were added, and 3D protonation and energy minimization was performed to further prepare the receptor proteins for molecular docking.

#### 2.7.3 MD simulation

Protein structures were modeled using BOLTZ-1 (Wohlwend et al., 2024; Passaro et al., 2025). The amino acid sequence of α-amylase (PDB ID: 1B2Y) and α-glucosidase (PDB ID: 2ZE0) were retrieved from the Protein Data Bank (PDB). On the other side, the SMILES string was obtained by PubChem database, the ligands used for the modelling was Nardostachysin (CID: 10598736) and Virolin (CID: 6440407) respectively.

Before running the molecular dynamic simulations (MDS), the ligands were parametrized, and the assembly was set with the Amber forcefield FF19SB (Tian et al., 2020) to a cubic box under Physiological conditions (pH 7.4, salt concentration 0.15 M) with the Amber Tools v25 software (Case et al., 2023). Amber FF19SB forcefield was selected for its improved protein backbone parameters trained against quantum mechanical calculations, ensuring accurate representation of protein-ligand interactions in physiological conditions (Tian et al., 2020). The MDS was executed at a total time of 100 ns using the software OpenMM due to its GPU-accelerated performance and computational efficiency (Eastman et al., 2024; Eastman et al., 2017) with Periodic Boundary Conditions (PBC). The parameters of the simulation consisted of a temperature of 300 K, for NPT equilibration we used the Monte Carlo Barostat. Finally, the MDS were analyzed using the MD Analysis software version 2.9.0 (Michaud‐Agrawal et al., 2011).

#### 2.7.4 Density functional theory calculations

For lead compounds against each protein, all quantum chemical calculations were performed using Gaussian 09 at the B3LYP/6-311G++(d,p) level of theory (Rahman et al., 2019). Molecular geometries were fully optimized in aqueous solution using the polarizable continuum model (PCM) with water as solvent (ε = 78.35). Frequency calculations confirmed all optimized structures as true minima with no imaginary frequencies. Electronic properties including frontier molecular orbital energies, dipole moments, and atomic charges were extracted from the converged wavefunctions. Global reactivity descriptors were calculated according to conceptual density functional theory: ionization potential (I = -EHOMO), electron affinity (A = -ELUMO), chemical hardness (η = (I-A)/2), chemical softness (S = 1/2η), electronegativity (χ = (I + A)/2), and electrophilicity index (ω = χ^2^/2η).

#### 2.7.5 Drug scanning through pharmacokinetics parameters

Assessing druglikness is a crucial step in identifying potential drug candidates with drug-like behavior. To evaluate the drug-likness of the top phytochemicals, SwissADME was employed (Daina et al., 2017). Furthermore, an online server called ADMETLab 3.0 was utilized to perform a comprehensive assessment of pharmacokinetics and pharmacodynamics properties. The ADMET profiling involved checking various parameters, including blood-brain barrier permeability, carcinogenicity, human intestinal absorption, Ame’s toxicity, and CaCo-2 permeability. These assessments help predict the drug-like behavior of a candidate from a clinical biochemistry perspective.

In addition to the ADMET profiling, the compounds were tested for Lipinski’s rule of five (Ro5) compliance. According to Lipinski’s rule, a drug candidate should have a molecular mass below 500 g/mol, no more than five hydrogen bond donors, no more than ten hydrogen bond acceptors, a logP value of less than or equal to five, and a molecular refractivity index within the range of 40–130 (Ahmad et al., 2023). If the compounds successfully met all these parameters, they could be considered potential leading drug candidates and might be further processed for development as potential treatments for diabetes. The drug-likeness properties of the selected compound were evaluated using the MolSoft online server (Sampat et al., 2022). Preliminary toxicity profiling was performed with StopTox (Borba et al., 2022), and further *In silico* toxicity assessment was carried out using ProTox 3.0, a predictive platform for chemical toxicity screening (Banerjee et al., 2024).

## 3 Results

### 3.1 % yield of total plant extract

Soxhlet extraction of *N. jatamansi* yielded 1.05 ± 0.02 g from 10.00 ± 0.01 g starting material, corresponding to 10.5% ± 0.2% w/w recovery from the initial plant material. Collective % yield was higher than individual rhizome percentage yield that is already reported 9% (Lyle et al., 2009). The extract was dark green in appearance.

### 3.2 Enzymatic assay-based inhibition

**FIGURE 2:**
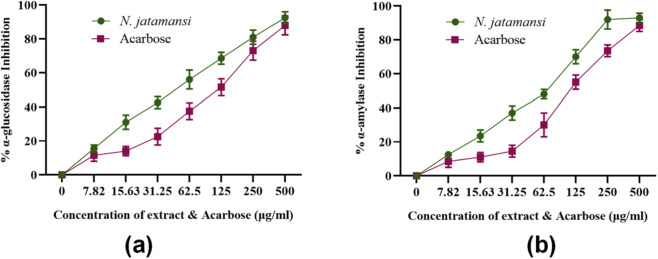
Dose-response curves showing percentage inhibition of **(a)** α-glucosidase and **(b)** α-amylase by *N. jatamansi* extract and acarbose (*n* = 3).

The inhibitory potential of *N. jatamansi* extract against carbohydrate-hydrolyzing enzymes was evaluated through *in vitro* enzymatic assays. The extract demonstrated dose-dependent inhibition of both α-glucosidase and α-amylase activities, with distinct potency profiles for each enzyme (Figure 2). The dose-response curves revealed sigmoidal relationships between extract concentration (0–500 μg/mL) and percentage enzyme inhibition. For α-glucosidase inhibition, *N. jatamansi* extract exhibited rapid initial response at lower concentrations (0–100 μg/mL), achieving approximately 50% inhibition at 61.7 ± 3.9 μg/mL, with gradual saturation approaching 90% inhibition at 500 μg/mL (Figure 2a). The α-amylase inhibition profile demonstrated a similar pattern but required higher concentrations, reaching 50% inhibition at 81.3 ± 4.7 μg/mL and maximum inhibition of approximately 92% at 500 μg/mL (Figure 2b).

**FIGURE 3:**
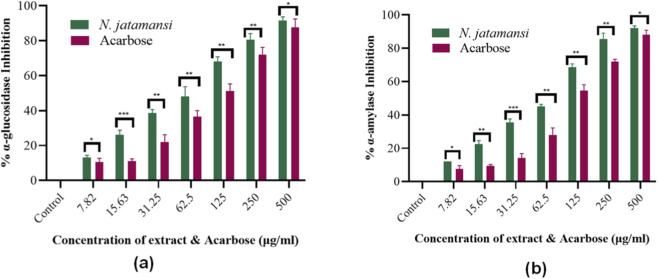
Dose-response inhibition profiles of *N. jatamansi* extract and acarbose against carbohydrate hydrolyzing enzymes. **(a)** α-glucosidase inhibition and **(b)** α-amylase inhibition by *N*. *jatamansi* extract (green bars) and acarbose (red bars) at concentrations ranging from 7.82 to 500 μg/mL. Data represent mean ± standard deviation (SD) from three independent experiments (*n* = 3) performed in triplicate. Statistical significance between *N*. *jatamansi* and acarbose at each concentration was determined using unpaired Student’s t-test. *p ≤ 0.05, **p ≤ 0.01, ***p ≤ 0.001. Control represents enzyme activity in the absence of inhibitors (0% inhibition baseline).

Comparative analysis with the standard inhibitor acarbose revealed superior potency of *N. jatamansi* extract against both enzymes (Table 1; Figure 3). The extract demonstrated 1.8-fold greater potency than acarbose against α-amylase (IC_50_: 81.3 ± 4.7 μg/mL vs. 112.1 ± 6.2 μg/mL) and 2.1-fold enhanced activity against α-glucosidase (IC_50_: 61.7 ± 3.9 μg/mL vs. 132.6 ± 7.8 μg/mL). The lower IC_50_ values for *N. jatamansi* extract indicate more effective enzyme inhibition at lower concentrations compared to the clinical standard.

**TABLE 1:** The IC_50_ values of *N. jatamansi* extract for α-glucosidase and α-amylase.

Sample	α-amylase IC_50_ (μg/mL)	α-glucosidase IC_50_ (μg/mL)
*N. jatamansi*	81.3 ± 4.7	61.7 ± 3.9
Acarbose	112.1 ± 6.2	132.6 ± 7.8

The extract displayed preferential inhibition toward α-glucosidase compared to α-amylase, with approximately 24% lower IC_50_ value for α-glucosidase. This selective inhibition profile suggests potential advantages in postprandial glucose management, as stronger α-glucosidase inhibition relative to α-amylase may reduce gastrointestinal side effects associated with undigested carbohydrate fermentation.

The consistent inhibitory activity across multiple enzyme assay replicates, as indicated by the relatively low standard deviation values range (±3.9 to ±7.8 μg/mL), confirms the reproducibility and reliability of the observed effects. These findings establish *N. jatamansi* extract as a potent dual inhibitor of key carbohydrate-metabolizing enzymes, warranting further investigation of its bioactive constituents through molecular level analyses.

### 3.3 Molecular docking results

#### 3.3.1 Compound selection from virtual screening

Molecular docking of the complete phytochemical library comprising 144 bioactive compounds from *N. jatamansi* was performed simultaneously against both α-glucosidase and α-amylase. The docking results revealed that previously reported compounds including Linarin (PubChem ID: 5317025) (Chenafa et al., 2022), Pinoresinol (PubChem ID: 73399) (Kwon et al., 2014), Ursolic acid (PubChem ID: 64945) (Wu et al., 2017), Valerenic acid (PubChem ID: 6440940) (Maurya et al., 2023), and Ferulic acid (PubChem ID: 445858) (Zheng et al., 2020) ranked among the top hits, demonstrating strong binding energies exceeding −9.5 kcal/mol with up to five conventional hydrogen bonds. However, since these compounds have been extensively characterized for their antidiabetic properties in literature, they were excluded from further analysis. Instead, the present investigation focused on novel, unexplored sesquiterpenoids from *N. jatamansi* to identify new therapeutic candidates with potential antidiabetic activity.

#### 3.3.2 α-glucosidase inhibition

**FIGURE 4:**
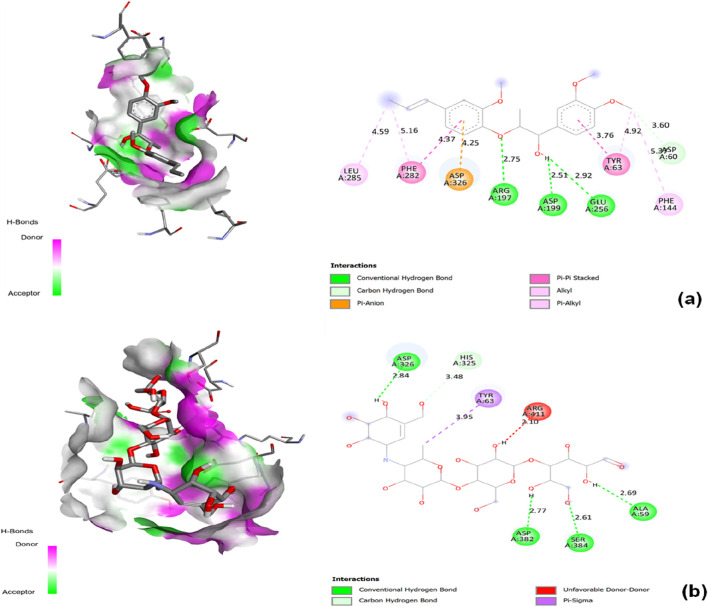
2D Interaction and 3D binding pattern of **(a)** Virolin **(b)** Acarbose with α-glucosidase as a receptor.

Analysis of novel compounds against α-glucosidase (PDB ID: 2ZE0) identified five lead molecules with binding affinities ranging from −8.1 to −9.6 kcal/mol (Table 2). Virolin emerged as the most potent predicted α-glucosidase binder, exhibiting the highest binding affinity of −9.6 kcal/mol, surpassing the standard inhibitor acarbose (−8.6 kcal/mol) by 1.0 kcal/mol. Comprehensive interaction mapping revealed Virolin’s superior binding profile through a sophisticated multi-modal interaction network. The compound established three critical conventional hydrogen bonds with catalytic residues: ARG197 (2.75 Å), ASP199 (2.51 Å), and GLU256 (2.92 Å), directly engaging the enzyme’s catalytic machinery. The aromatic phenyl ring of Virolin participated in dual π-π stacked interactions with PHE282 at two distinct geometric orientations (5.16 Å and 4.37 Å), creating a stable aromatic sandwich complex. Similarly, TYR63 engaged in dual π-π stacking with Virolin’s aromatic system at 3.76 Å and 4.92 Å, further stabilizing the ligand within the binding pocket. A unique π-anion interaction between Virolin’s aromatic ring and the negatively charged ASP326 (4.25 Å) contributed electrostatic stabilization, while LEU285 formed a π-alkyl contact (4.59 Å) and PHE144 established an alkyl interaction (5.39 Å), anchoring the compound through hydrophobic forces. Additionally, a carbon-hydrogen bond with ASP60 (3.60 Å) provided supplementary stabilization. This intricate binding architecture, combining strong hydrogen bonding with extensive aromatic stacking and hydrophobic anchoring, explains Virolin’s superior computational binding potential over acarbose (Figure 4a).

**TABLE 2:** Binding scores of top five compounds with α-glucosidase (2ZE0) and α-amylase (1B2Y) protein receptor.

Name	PubChem ID	Binding affinity (kcal/mol) with 2ZE0	Interacting with amino acids with 2ZE0	Binding affinity (kcal/mol) with 1B2Y	Interacting with amino acids with 1B2Y
Acarbose 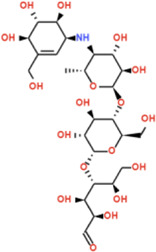	9811704	−8.6	ASP-324, HIS325, TYR63, ASP-382, SER-384 and ALA-59	−7.1	ARG-421, ARG-10, GLY-9, GLN-7, THR-6, SER-3, GLN-8, GLY-334 and PRO-332
Virolin 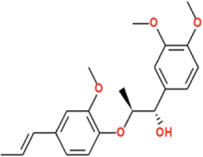	6440407	−9.6	LEU-285, PHE-282, ASP-326, ARG-197, ASP-199, GLU-256, TYR-63, ASP-60 and PHE-144	−7.1	TRP-59, HIS-201, ILE-235, ALA-198, LEU-162 and TYR-62
Nardostachysin 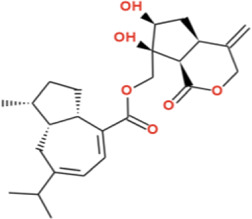	10598736	−9.0	PHE-144, HIS-325, ASP-326, TYR-63 and GLU-256	−9.5	TRP-59, LEU-165, HIS-305 and ASP-300
Nardosinonediol 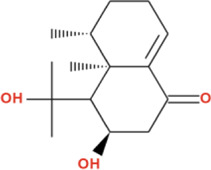	134715257	−8.6	PHE-163, ALA-200, HIS-103, TYR-63 and GLU-256	−9.0	HIS-305, TRP-59, GLN-63 and TYR-62
Bulnesol 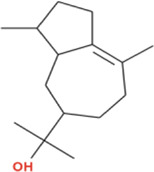	12302134	−8.3	PHE-144, HIS-103, PHE-163, ALA-200, TYR-63, ASN-61 and ARG-411	−8.4	TRP-59, ASP-197, TRP-58 and TYR-62
Oroselol 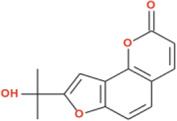	160600	−8.1	ALA-200, ASP-199, TYR-63 and PHE-163	−8.5	TYR-62, TRP-59, HIS-305, LEU-165 and ASP-300

^a^ (Structures of compounds inserted in Table 2 were retrieved from PubChem database).

In comparison, acarbose demonstrated a more conventional carbohydrate-mimetic binding profile with α-glucosidase (−8.6 kcal/mol). While acarbose formed four conventional hydrogen bonds with ASP326 (2.84 Å), ASP382 (2.77 Å), SER384 (2.61 Å), and ALA59 (2.69 Å), alongside a carbon-hydrogen bond with HIS325 (3.48 Å) and a π-sigma interaction with TYR63 (3.95 Å), it lacked the extensive aromatic stacking network observed with Virolin. Notably, acarbose exhibited an unfavorable donor-donor interaction with ARG411 (2.10 Å), which likely destabilizes the complex and contributes to its lower binding affinity. The absence of π-π stacking and π-anion interactions, coupled with this unfavorable contact, explains why acarbose exhibits reduced computational binding strength compared to Virolin despite forming multiple hydrogen bonds. Virolin’s sesquiterpenoid scaffold thus represents a structurally distinct candidate molecule that achieves superior binding through aromatic-dominated interactions rather than relying solely on hydrogen bonding networks characteristic of carbohydrate-based inhibitors (Figure 4b).

**FIGURE 5:**
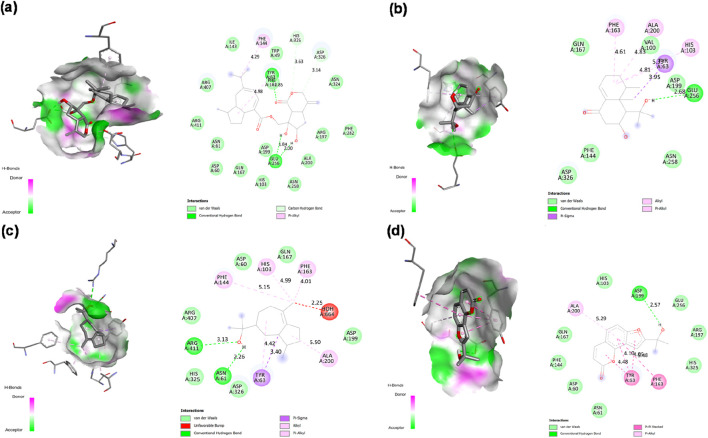
2D Interaction and 3D binding pattern with α-glucosidase as a receptor **(a)** Nardostachysin, **(b)** Nardosinonediol, **(c)** Bulnesol and **(d)** Oroselol.

Nardostachysin demonstrated the second-highest binding affinity (−9.0 kcal/mol) against α-glucosidase. Detailed interaction analysis revealed that the compound formed dual conventional hydrogen bonds with the catalytic residue GLU256 at 1.84 Å and 2.00 Å, representing exceptionally strong electrostatic interactions critical for enzyme inhibition. An additional conventional hydrogen bond with TYR63 (2.85 Å) further anchored the ligand within the active site. Carbon-hydrogen bonds with HIS325 (3.63 Å) and ASP326 (3.14 Å) provided supplementary polar stabilization near the catalytic pocket. The sesquiterpene scaffold established π-alkyl interactions with aromatic residues PHE144 (4.29 Å) and PHE163 (4.98 Å), creating favorable hydrophobic contacts that enhanced binding affinity. Van der Waals forces with surrounding residues including ARG407, ARG411, ARG197, ASN61, ASN258, ASN324, ASP60, ASP199, GLN167, HIS103, ALA200, ILE143, TRP49, and PHE282 enveloped the ligand throughout the binding pocket, providing comprehensive distributed stabilization. The combination of dual strong hydrogen bonds with the catalytic GLU256, additional polar interactions, strategic π-alkyl contacts, and extensive van der Waals network explains the high binding affinity, positioning Nardostachysin as the second most promising compound against α-glucosidase after Virolin (Figure 5a).

Nardosinonediol displayed moderate binding potential with a binding energy of −8.6 kcal/mol. The compound established a critical conventional hydrogen bond with the catalytic residue GLU256 (2.68 Å), directly engaging the enzyme’s catalytic machinery. The bicyclic sesquiterpene scaffold demonstrated extensive aromatic and hydrophobic interactions: dual π-sigma interactions with TYR63 at 4.81 Å and 3.95 Å provided favorable geometric orientation within the binding pocket. Multiple π-alkyl interactions dominated the hydrophobic contacts, including PHE163 (4.61 Å), ALA200 (4.83 Å), and HIS103 (5.39 Å), effectively filling the hydrophobic cavity and stabilizing the complex through aromatic-aliphatic contacts. Van der Waals forces with surrounding residues including GLN167, PHE144, ASP326, ASP199, ASN258, and VAL100 created a distributed interaction network that enveloped the ligand within the active site. The combination of direct hydrogen bonding with the catalytic GLU256, dual π-sigma interactions with TYR63, and extensive π-alkyl contacts explains the moderate binding affinity, positioning Nardosinonediol as a viable lead candidate despite lower binding energy compared to Virolin and Nardostachysin (Figure 5b).

Bulnesol exhibited a binding affinity of −8.3 kcal/mol with a distinctive interaction profile. The compound formed conventional hydrogen bonds with ARG411 (3.13 Å) and ASN61 (2.26 Å), positioning it within the active site pocket. However, an unfavorable bump interaction with HOH A:664 (2.25 Å) was detected, representing a steric clash that may destabilize the complex and limit overall binding strength. Despite this unfavorable contact, the tricyclic sesquiterpene scaffold compensated through extensive aromatic and hydrophobic interactions: dual π-sigma interactions with TYR63 at 4.42 Å and 3.40 Å provided favorable geometric orientation. Multiple π-alkyl contacts dominated the hydrophobic binding network, including PHE144 (5.15 Å), HIS103 (4.99 Å), and PHE163 (4.01 Å), effectively occupying the hydrophobic cavity. An alkyl interaction with ALA200 (5.50 Å) further enhanced hydrophobic stabilization. Van der Waals forces with surrounding residues including ARG407, ASP60, HIS325, ASP326, GLN167, and ASP199 provided distributed stabilization throughout the binding pocket. The balance between the unfavorable water-mediated clash and the extensive aromatic/hydrophobic interaction network resulted in moderate binding affinity, positioning Bulnesol as a potential lead candidate despite its slightly lower binding energy (Figure 5c).

Oroselol maintained stable interactions with a binding affinity of −8.1 kcal/mol, representing the lowest binding energy among the five lead candidates. The compound established a conventional hydrogen bond with the catalytic residue ASP199 (2.57 Å), directly engaging the enzyme’s catalytic machinery and anchoring the ligand within the active site. Aromatic interactions dominated the binding profile: triple π-π stacked interactions with TYR63 at 4.48 Å, 4.10 Å, and 4.05 Å provided extensive aromatic stabilization through electronic complementarity, while an additional π-π stacked interaction with PHE163 (3.48 Å) further enhanced the aromatic binding network. A π-alkyl interaction with ALA200 (5.29 Å) contributed to hydrophobic stabilization. Van der Waals forces with surrounding residues including HIS103, GLU256, ARG197, HIS325, PHE144, ASP60, ASN61, and GLN167 created a distributed interaction network throughout the binding pocket. The combination of direct hydrogen bonding with the catalytic ASP199 and multiple π-π stacked interactions with aromatic residues explains the moderate binding affinity. Despite exhibiting the lowest binding energy among the five candidates, Oroselol’s strategic engagement with catalytic residues through both polar and extensive aromatic interactions suggests potential as a lead scaffold for further optimization (Figure 5d).

#### 3.3.3 α-amylase activity

**FIGURE 6:**
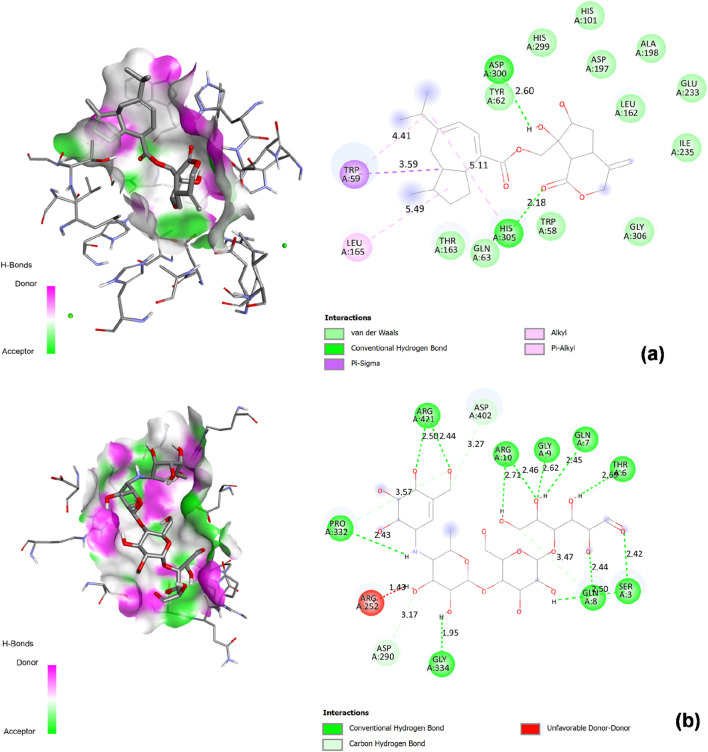
2D Interaction and 3D binding pattern of **(a)** Nardostachysin **(b)** Acarbose with α-amylase as a receptor.

Molecular docking against α-amylase (PDB ID: 1B2Y) identified Nardostachysin as the lead compound with the highest binding affinity of −9.5 kcal/mol (Table 2), demonstrating a remarkable 2.4 kcal/mol superior binding energy compared to the clinical standard acarbose (−7.1 kcal/mol). This substantial energetic advantage translates to significantly enhanced predicted binding stability and catalytic site occupancy. Comprehensive interaction mapping revealed that Nardostachysin’s sesquiterpenoid scaffold strategically engaged the catalytic machinery through dual hydrogen bonding interactions with critical active site residues: ASP300 (2.60 Å) and HIS305 (2.18 Å). ASP300, a member of the catalytic triad essential for glycosidic bond hydrolysis, forms a particularly strong hydrogen bond at optimal geometry, while HIS305 provides secondary catalytic support. Beyond hydrogen bonding, Nardostachysin exhibited sophisticated aromatic interactions with TRP59 through both π-sigma (3.59 Å) and π-alkyl (4.41 Å) contacts at distinct molecular orientations, creating a dual-mode aromatic engagement that anchors the compound within the binding cleft. Additional π-alkyl interactions with HIS305 (5.11 Å) and LEU165 (5.49 Å) provided extensive hydrophobic stabilization, effectively filling the substrate-binding groove and blocking access to the catalytic center. This balanced combination of strong catalytic residue hydrogen bonding with extensive aromatic and hydrophobic interactions explains Nardostachysin’s exceptional computational binding affinity and predicted enzyme blocking capacity (Figure 6a).

In stark contrast, acarbose demonstrated a hydrogen bond-dominated binding profile with α-amylase (−7.1 kcal/mol), forming an extensive network of eleven conventional hydrogen bonds spanning multiple subsites: PRO332 (2.43 Å, 3.57 Å), GLY334 (1.95 Å), GLN50 (3.47 Å, 2.44 Å), SER8 (2.42 Å), THR6 (2.65 Å), GLN7 (2.45 Å), GLY9 (2.62 Å), ARG10 (2.46 Å, 2.71 Å), and ARG421 (2.50 Å, 2.44 Å), alongside a carbon-hydrogen bond with ASP402 (3.27 Å). Despite this impressive hydrogen bonding network characteristic of its pseudotetrasaccharide structure, acarbose exhibited 2.4 kcal/mol lower binding affinity than Nardostachysin. The critical difference lies in interaction quality rather than quantity: acarbose’s hydrogen bonds predominantly engaged peripheral binding subsite residues rather than directly contacting the catalytic triad with the same geometric precision observed for Nardostachysin. Furthermore, acarbose completely lacked the aromatic π-interactions (π-sigma, π-π stacking, π-alkyl) that provide Nardostachysin with deep catalytic pocket penetration and enhanced binding stability. This comparison reveals that sesquiterpenoid scaffolds can achieve superior computational binding through targeted catalytic engagement and aromatic-hydrophobic synergy, rather than relying solely on multiple polar contacts distributed across binding subsites (Figure 6b).

**FIGURE 7:**
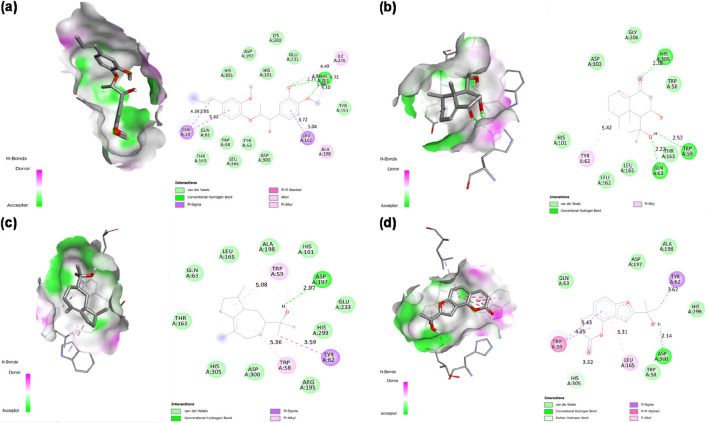
2D Interaction and 3D binding pattern with α-amylase as a receptor **(a)** Virolin, **(b)** Nardosinonediol, **(c)** Bulnesol and **(d)** Oroselol.

Nardosinonediol exhibited substantial predicted binding potential with an affinity of −9.0 kcal/mol, ranking as the second-strongest binder against α-amylase and demonstrating 1.9 kcal/mol superior binding energy compared to acarbose (−7.1 kcal/mol). The compound established a focused hydrogen bonding network with three strategic residues: HIS305 (2.18 Å), a catalytic site component; TRP59 (2.53 Å), a critical substrate recognition residue; and GLN63 (2.22 Å), which stabilizes the binding pocket conformation. These short-distance hydrogen bonds indicate optimal geometric complementarity with the active site architecture. Additionally, the bicyclic sesquiterpene scaffold formed a π-alkyl interaction with TYR62 (5.42 Å), providing hydrophobic anchoring within the binding groove. The compact binding profile, characterized by precise catalytic residue engagement through strong hydrogen bonds, explains Nardosinonediol’s high computational affinity and suggests efficient active site blockage despite forming fewer total interactions than larger polycyclic compounds (Figure 7b).

Oroselol demonstrated favorable binding affinity of −8.5 kcal/mol, exceeding acarbose by 1.4 kcal/mol and representing a promising candidate structure. The compound’s binding mode combined catalytic engagement through a conventional hydrogen bond with ASP300 (2.14 Å), a key catalytic triad residue, with extensive aromatic interactions. TRP59 participated in dual π-π stacked interactions at two distinct geometric orientations (4.35 Å and 5.43 Å), creating a stable sandwich complex that anchors Oroselol deep within the substrate-binding pocket. A π-sigma interaction with TYR62 (3.62 Å) and π-alkyl contact with LEU165 (5.31 Å) provided additional hydrophobic stabilization. This balanced interaction profile, combining direct catalytic site hydrogen bonding with strong aromatic stacking, explains Oroselol’s competitive computational binding energy and suggests effective active site occupancy through a compact molecular architecture (Figure 7d).

Bulnesol showed moderate predicted binding affinity of −8.4 kcal/mol, demonstrating 1.3 kcal/mol enhanced binding compared to acarbose. The compound established a single critical hydrogen bond with ASP197 (2.97 Å), a catalytic triad residue involved in substrate hydrolysis. The tricyclic sesquiterpene scaffold engaged in extensive aromatic and hydrophobic interactions: TRP59 formed a π-alkyl contact (5.08 Å), TRP58 provided additional π-alkyl stabilization (5.36 Å), and TYR62 contributed through π-sigma interaction (3.59 Å). This predominantly hydrophobic binding mode, with aromatic residue engagement across the substrate-binding groove, effectively fills the active site cavity. The reliance on a single strong hydrogen bond supplemented by multiple aromatic contacts represents an alternative binding strategy compared to the dual hydrogen bonding observed in higher-affinity compounds (Figure 7c).

Virolin, despite being the lead compound for α-glucosidase (−9.6 kcal/mol), exhibited the lowest binding affinity against α-amylase at −7.1 kcal/mol, equivalent to acarbose and demonstrating clear enzyme selectivity. The compound formed a single weak conventional hydrogen bond with HIS201 (1.99 Å), which, while geometrically short, does not directly engage the catalytic triad (ASP197, GLU233, ASP300). The binding profile was dominated by multiple π-sigma interactions with TRP59 at three distinct orientations (4.38 Å, 3.85 Å, 5.82 Å) and LEU162 (3.72 Å), alongside π-alkyl contacts with ILE235 at four positions (4.49 Å, 5.10 Å, 4.72 Å, 4.98 Å) and ALA198 (5.04 Å). This purely peripheral binding mode, lacking strong catalytic residue hydrogen bonding and relying entirely on diffuse hydrophobic interactions, explains the reduced affinity compared to other sesquiterpenoids tested. The dramatic difference in binding energy between α-glucosidase (−9.6 kcal/mol) and α-amylase (−7.1 kcal/mol) reveals Virolin’s enzyme-selective binding driven by differential active site architecture recognition (Figure 7a).

#### 3.3.4 Virtual screening and identification of promising hits

Based on the comprehensive docking analysis, Virolin was identified as the Promising hit for α-glucosidase with strong binding affinity (−9.6 kcal/mol), while Nardostachysin emerged as the most potent α-amylase potential inhibitor (−9.5 kcal/mol). Interestingly, Nardostachysin demonstrated dual binding potential with high binding affinities against both enzymes (−9.0 kcal/mol for α-glucosidase and −9.5 kcal/mol for α-amylase), suggesting its potential as a dual-target antidiabetic agent. These distinct binding profiles and enzyme selectivity’s provide valuable insights for developing targeted therapeutic interventions from *N. jatamansi* phytochemicals.

### 3.4 Molecular dynamics simulation analysis

#### 3.4.1 Validation of docking predictions through dynamic simulations

To validate the binding modes and stability of the top-ranked compounds identified through molecular docking, 100 ns molecular dynamics simulations were performed for the promising compound identified through virtual screening: Nardostachysin with α-amylase (PDB ID: 1B2Y) and Virolin with α-glucosidase (PDB ID: 2ZE0). These simulations provided insights into the dynamic behaviour, conformational stability, and binding energetics of the protein-ligand complexes under physiological conditions.

#### 3.4.2 Structural stability and conformational dynamics

**FIGURE 8:**
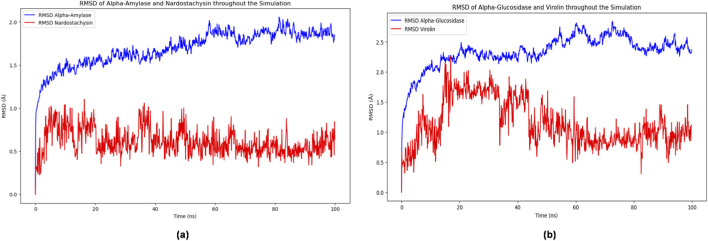
RMSD plots of α-amylase with Nardostachysin **(a)** and α-glucosidase with Virolin **(b)** during 100 ns molecular dynamics simulation.

The root mean square deviation (RMSD) analysis revealed distinct stability profiles for both complexes (Figure 8). The α-amylase-Nardostachysin complex demonstrated solid structural stability throughout the simulation trajectory. Following an initial equilibration phase during the first 20 ns, the protein backbone achieved convergence with an average RMSD of 1.8 Å, indicating minimal structural reorganization and robust complex formation. Remarkably, Nardostachysin maintained extraordinary conformational rigidity within the active site, with ligand RMSD values consistently below 1.0 Å throughout the simulation, suggesting optimal positioning and stable molecular anchoring.

In contrast, the α-glucosidase Virolin complex exhibited greater conformational dynamics. The protein structure displayed higher fluctuations with an average RMSD of 2.5 Å, indicating enhanced structural plasticity and continuous adaptation of the protein domains. Virolin demonstrated considerably more mobility within the binding pocket, with erratic RMSD fluctuations and periodic peaks, reflecting reduced positional stability compared to the Nardostachysin complex.

#### 3.4.3 Residue flexibility and local dynamics

**FIGURE 9:**
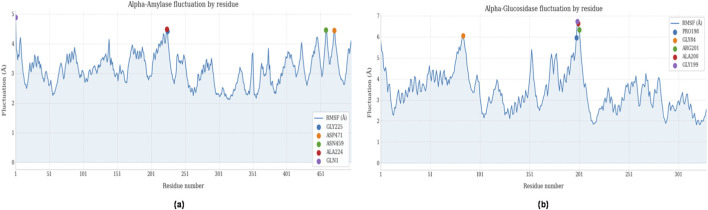
RMSF plots during 100 ns simulation: **(a)** α-amylase by residue, **(b)** α-glucosidase by residue.

Root mean square fluctuation (RMSF) analysis identified regions of flexibility within both protein structures (Figure 9). In α-amylase, residues ALA224, ASP471, and ASN459 exhibited maximum fluctuations of approximately 4.5 Å, corresponding to flexible loops and surface regions that may indirectly influence active site conformation. The N-terminal residue GLN1 showed the highest flexibility (∼5.0 Å), characteristic of terminal chain dynamics.

The α-glucosidase structure displayed enhanced flexibility in the region encompassing PRO198, GLY199, ALA200, and ARG201, with RMSF values reaching 6.5 Å. This elevated mobility near the binding site suggests reduced structural compaction and potential active reorganization during ligand interaction, which may contribute to the lower binding stability observed for Virolin.

#### 3.4.4 Binding energetics and thermodynamic stability

**FIGURE 10:**
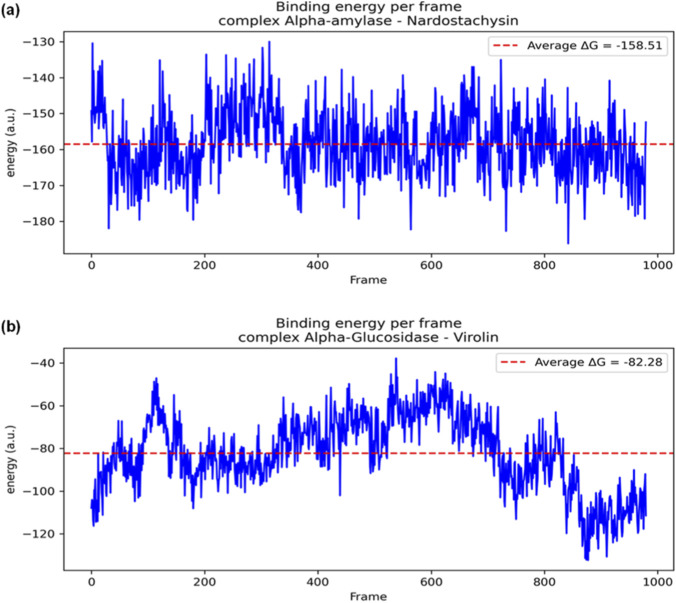
Binding energy profiles per frame during 100 ns simulation: **(a)** α-amylase-Nardostachysin complex, **(b)** α-glucosidase-Virolin complex.

The binding free energy calculations revealed substantial differences in the thermodynamic favourability of the two complexes (Figure 10). The α-amylase Nardostachysin complex maintained an average binding free energy (ΔG) of −158.51 kcal·mol^−1^ throughout the simulation, indicating highly favorable protein-ligand interactions. This substantial negative energy value correlates with the special stability observed in the RMSD analysis and confirms the strong binding affinity predicted by molecular docking (−9.5 kcal/mol).

The α-glucosidase Virolin complex demonstrated a significantly less favorable binding energy of ΔG = −82.28 kcal·mol^−1^. This difference of approximately 76 kcal·mol^−1^ between the two complexes highlights virolin’s comparatively lower thermodynamic stability despite its high docking score (−9.6 kcal/mol). The reduced binding energy corresponds with the increased conformational fluctuations observed in the RMSD analysis, suggesting that static docking scores may overestimate binding affinity without considering dynamic effects (Figure 10).

#### 3.4.5 Molecular interaction profiles

**FIGURE 11:**
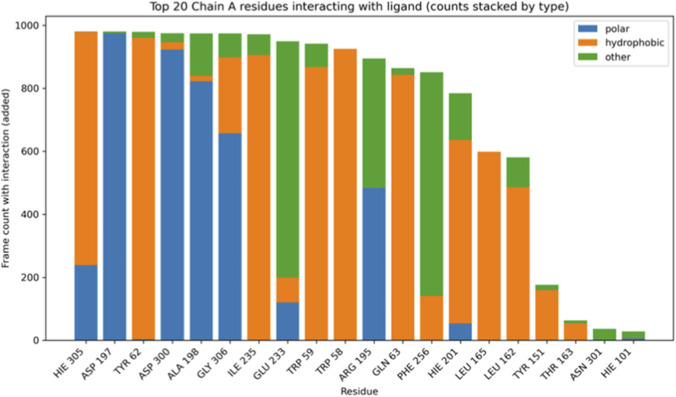
Top 20 residues of the A chain of α-amylase interacting with the ligand Nardostachysin (stacked count by type).

Analysis of protein-ligand interactions throughout the simulation revealed distinct binding patterns for each complex. In the α-amylase Nardostachysin complex, residues HIS305, ASP197, and TYR62 maintained the highest contact frequencies throughout the trajectory (Figure 11). HIS305 and TYR62 predominantly contributed hydrophobic interactions, while ASP197 established crucial polar contacts. This balanced combination of interaction types, maintaining both high frequency and energetic diversity, substantiates the robust anchoring observed in structural analyses.

**FIGURE 12:**
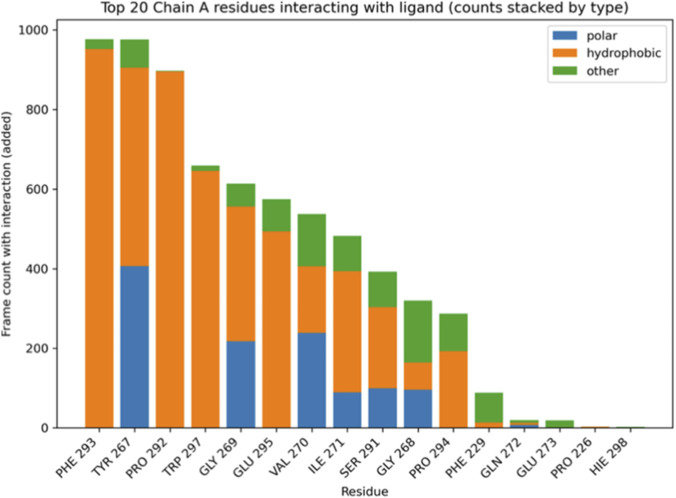
Top 20 residues of the α-glucosidase A chain interacting with the Virolin ligand (stacked count by type).

The α-glucosidase Virolin complex interaction profile was dominated by PHE293, TYR292, and PRO292, with hydrophobic interactions being overwhelmingly predominant (Figure 12). Despite high contact frequencies in the temporal stack, the less diverse interaction profile and lower binding energy suggest these hydrophobic contacts alone are insufficient to maintain complex stability comparable to the mixed interaction network in the Nardostachysin complex.

#### 3.4.6 Critical distance analysis and complex integrity

**FIGURE 13:**
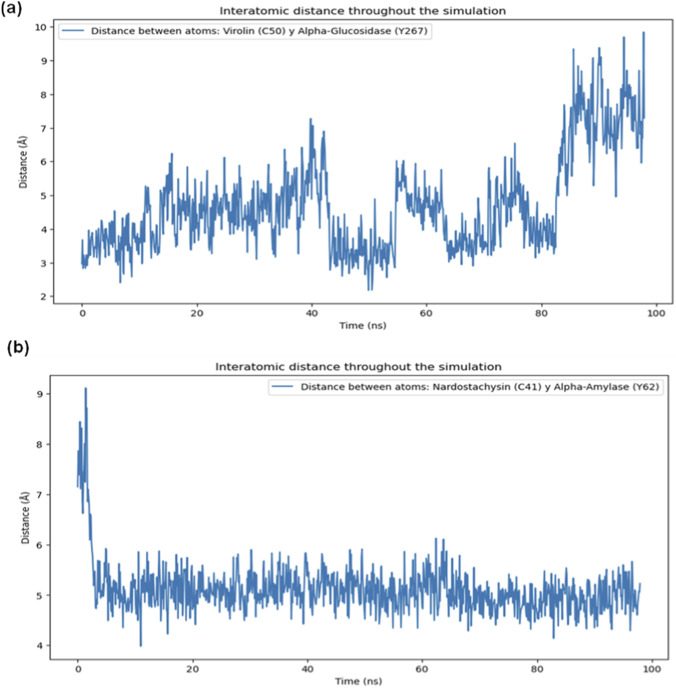
Interatomic distance profiles during 100 ns simulation: **(a)** Virolin with α-glucosidase (C50–Y267), **(b)** Nardostachysin with α-amylase (C41–Y62).

Monitoring of representative interatomic distances provided direct evidence of binding stability (Figure 13). The distance between C41 of Nardostachysin and TYR62 of α-amylase rapidly stabilized within a narrow range of 4.5–6.0 Å following equilibration. This consistent proximity throughout the 100 ns simulation confirms persistent ligand retention in an optimal binding orientation without significant positional drift.

Conversely, the α-glucosidase Virolin complex displayed considerable distance fluctuations between C50 of Virolin and TYR267 of α-glucosidase. Initial oscillations between 2.5 and 6.0 Å during the first 75 ns were followed by a dramatic increase to 8–10 Å in the final simulation quarter. This late-stage distance expansion indicates an incipient dissociation event or substantial conformational rearrangement that compromises binding integrity, consistent with the lower binding energy and elevated RMSD values.

#### 3.4.7 Comparative assessment of binding stability

The molecular dynamics simulations provide compelling evidence for the superior stability of Nardostachysin as an α-amylase potential inhibitor compared to virolin’s interaction with α-glucosidase. Despite virolin’s marginally higher docking score, the dynamic simulations reveal that Nardostachysin forms a more thermodynamically stable complex with sustained molecular interactions and minimal conformational drift. These findings underscore the importance of complementing static docking analyses with dynamic simulations to accurately assess protein-ligand binding potential and identify truly effective enzyme inhibitors from *N. jatamansi*.

### 3.5 Density functional theory calculations

#### 3.5.1 Electronic properties and frontier molecular orbitals

The electronic properties of the lead compounds were systematically analyzed through DFT calculations to establish structure-activity relationships at the quantum mechanical level. Table 3 presents the calculated frontier molecular orbital energies and derived reactivity descriptors for both compounds.

**TABLE 3:** Electronic properties and reactivity descriptors of lead compounds.

Property	Virolin	Nardostachysin	Unit
Frontier molecular orbitals			
E(HOMO)	−5.85	−5.78	eV
E(LUMO)	−1.07	−1.21	eV
HOMO-LUMO gap (ΔE)	4.78	4.57	eV
Reactivity descriptors			
Ionization potential (I)	5.85	5.78	eV
Electron affinity (A)	1.07	1.21	eV
Chemical hardness (η)	2.39	2.29	eV
Chemical softness (S)	0.209	0.219	eV^-1^
Electronegativity (χ)	3.46	3.50	eV
Electrophilicity index (ω)	2.50	2.68	eV
Molecular properties			
Dipole moment (μ)	4.83	6.29	Debye
Polarizability (α)	375.78	364.19	Bohr^3^
Total energy	−1192.2714	−1424.494	Hartree

The frontier molecular orbital analysis revealed notable electronic characteristics distinguishing the two lead compounds. Virolin exhibited a HOMO energy of −5.85 eV and LUMO energy of −1.07 eV, resulting in a HOMO-LUMO energy gap of 4.78 eV. Nardostachysin demonstrated comparable orbital energies with EHOMO = −5.78 eV and ELUMO = −1.21 eV, yielding a slightly narrower energy gap of 4.57 eV. The 0.21 eV difference in energy gaps indicates that Nardostachysin possesses marginally enhanced electronic reactivity, which may contribute to its dual inhibitory capacity against both target enzymes.

Analysis of the reactivity descriptors calculated from conceptual density functional theory revealed distinct chemical behavior patterns. Virolin displayed higher chemical hardness (η = 2.39 eV) compared to Nardostachysin (η = 2.29 eV), indicating greater resistance to electronic deformation during chemical interactions. The corresponding chemical softness values were 0.209 eV^-1^ for Virolin and 0.219 eV^-1^ for Nardostachysin, confirming Nardostachysin’s enhanced reactivity profile. Both compounds demonstrated similar electronegativity values (χ = 3.46 eV for Virolin; χ = 3.50 eV for Nardostachysin), while the electrophilicity indices differed slightly (ω = 2.51 eV for Virolin; ω = 2.67 eV for Nardostachysin), suggesting Nardostachysin’s superior electron-accepting capacity.

The molecular polarity analysis revealed significant structural differences. Nardostachysin exhibited a substantially higher dipole moment (μ = 6.29 Debye) compared to Virolin (μ = 4.83 Debye), indicating enhanced molecular polarity that correlates with its observed dual predicted enzyme inhibitory activity. This increased polarity likely facilitates stronger electrostatic interactions with diverse amino acid residues across different enzyme active sites. Virolin demonstrated slightly higher polarizability (α = 375.78 Bohr^3^) compared to Nardostachysin (α = 364.19 Bohr^3^), suggesting greater capacity for induced dipole formation under the influence of external electric fields from enzyme residues.

#### 3.5.2 Molecular orbital visualization and electron distribution

**FIGURE 14:**
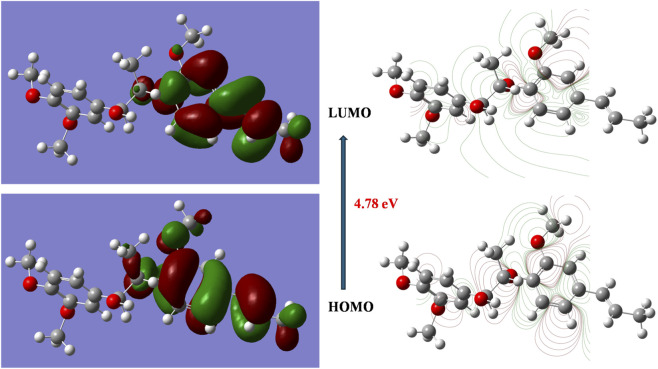
HOMO and LUMO molecular orbital surfaces of Virolin calculated at B3LYP/6–311++G(d,p) level in aqueous solution. Green and red surfaces represent positive and negative orbital phases respectively (isovalue = 0.02 a.u.).

The spatial distribution of electron density in the frontier molecular orbitals provides critical insights into the binding mechanisms observed in molecular docking studies. Figure 14 displays the HOMO and LUMO surfaces for Virolin, revealing concentrated electron density localization on aromatic ring systems and oxygen-containing functionalities in the HOMO distribution. These regions represent primary sites for electron donation during enzyme interactions, consistent with the hydrogen bonding patterns identified in the docking analysis with α-glucosidase residues.

**FIGURE 15:**
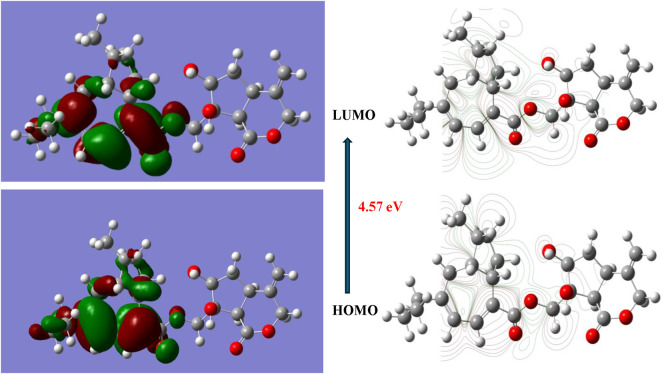
HOMO and LUMO molecular orbital surfaces of Nardostachysin calculated at B3LYP/6–311 ++G(d,p) level in aqueous solution. Green and red surfaces represent positive and negative orbital phases respectively (isovalue = 0.02 a.u.).

The LUMO surfaces of Virolin exhibit more diffuse character with significant contributions from aromatic π systems, indicating potential for π-π stacking interactions and charge transfer mechanisms with aromatic amino acid residues such as Phe282 and Phe144 observed in the binding studies. Similarly, Figure 15 presents the frontier orbital surfaces for Nardostachysin, showing comparable electron density distribution patterns but with enhanced delocalization corresponding to its higher dipole moment and broader enzyme selectivity.

#### 3.5.3 Molecular electrostatic potential and charge distribution

**FIGURE 16:**
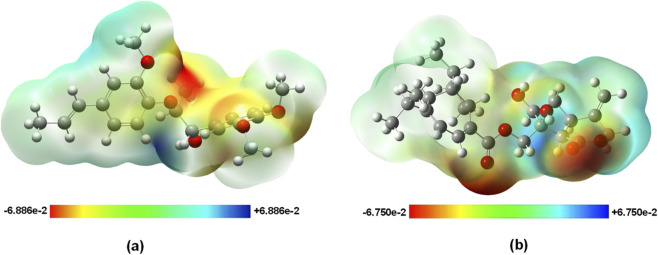
Molecular electrostatic potential surfaces mapped onto electron density isosurfaces of **(a)** Virolin and **(b)** Nardostachysin calculated at B3LYP/6–311++G(d,p) level in aqueous solution. Red regions indicate negative potential (electron-rich areas), while blue regions represent positive potential (electron-deficient areas). Color scales are shown in atomic units.

The molecular electrostatic potential analysis provides fundamental understanding of the charge distribution patterns governing enzyme recognition. Both compounds exhibited concentrated negative electrostatic potential regions around oxygen atoms, explaining their nucleophilic character and propensity for hydrogen bond formation with positively charged enzyme residues such as Arg197 and His305 identified in the molecular docking studies (Figure 16).

Nardostachysin’s elevated dipole moment (6.29 D) compared to Virolin (4.83 D) reflects its more polarized charge distribution, which correlates directly with its predicted dual inhibitory capacity against both α-amylase and α-glucosidase. This enhanced polarity enables Nardostachysin to adapt to the different electrostatic environments present in the distinct enzyme active sites, explaining its broader selectivity profile observed in the binding affinity studies.

The quantum mechanical calculations provide a fundamental electronic basis for understanding the structure-activity relationships governing sesquiterpenoid enzyme inhibition, establishing how intrinsic molecular electronic properties determine binding selectivity and inhibitory potency beyond simple structural complementarity.

### 3.6 Drug-likeness and ADMET profiling

**FIGURE 17:**
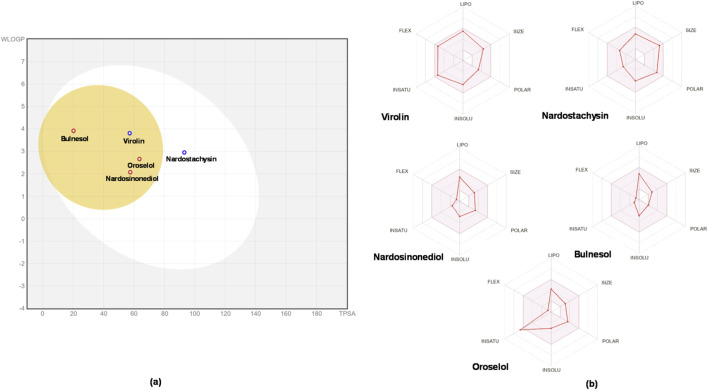
**(a)** BOILED-Egg plot showing predicted gastrointestinal absorption (white region) and blood–brain barrier permeability (yellow region) of the five lead compounds based on WLOGP and TPSA parameters. **(b)** Radar plots illustrate key physicochemical properties of each compound, including lipophilicity (LIPO), size, polarity (POLAR), insolubility (INSOLU), flexibility (FLEX), and unsaturation (INSATU), providing an overview of their drug-likeness profiles.

**FIGURE 18:**
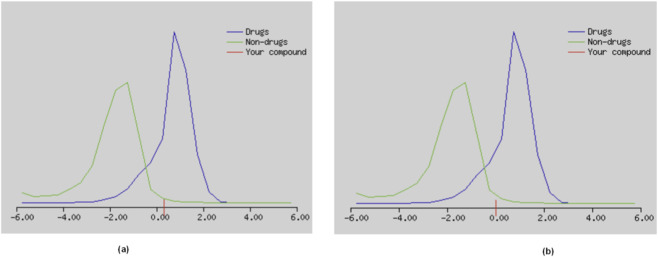
Molsoft drug-likeness score visualization: **(a)** Virolin (score: 0.33), **(b)** Nardostachysin (score: 0.01).

The drug-likeness and ADMET properties of the five top-ranked compounds were comprehensively evaluated (Tables 4–6; Figures 17, 18). All compounds demonstrated complete compliance with Lipinski’s Rule of Five, indicating favorable oral bioavailability potential (Table 4). Molecular weights ranged from 222.37–430.53 Da, hydrogen bond donors (1–2), acceptors (1–6), and lipophilicity values (LogP 2.11–3.41) all remained within acceptable limits.

**TABLE 4:** Molecular and drug likeliness properties of top 05 compounds evaluated through Lipinski’s Rule of Five.

Molecular properties							
Ligand	Molecular Mass (≤500 Da)	Hydrogen bond donor (≤5)	Hydrogen bond acceptor (≤10)	No. of rotatable bond (≤10)	Log P (≤5)	Refractivity (40–130)	Violations
Virolin	358.43	1	5	8	2.772	102.57	0
Nardostachysin	430.53	2	6	5	3.03	117.34	0
Nardosinonediol	252.35	2	3	1	2.11	71.82	0
Bulnesol	222.37	1	1	1	3.41	70.72	0
Oroselol	244.24	1	4	1	2.28	67.88	0

ADMET analysis revealed distinct pharmacokinetic profiles (Table 5). Blood-brain barrier penetration was highest for bulnesol (+++), while other compounds showed negligible permeability (---). Human intestinal absorption and Caco-2 permeability were uniformly low across all compounds. Volume of distribution values varied considerably (virolin: 0.305 L/kg; oroselol: −0.223 L/kg), with plasma protein binding ranging from 57.1% (nardosinonediol) to 96.3% (bulnesol).

**TABLE 5:** ADMET-related drug-like parameters of the best selected compounds.

Compounds					
​	Virolin	Nardostachysin	Nardosinonediol	Bulnesol	Oroselol
Absorption and distribution					
BBB	−−−	−−−	−−−	+++	−−−
HIA	−−−	−−−	−−−	−−−	−−−
Caco-2 permeability	−5.023	−4.995	−4.842	−4.7	−4.729
Volume of distribution (Vd)	0.305	0.089	−0.016	0.008	−0.223
Plasma protein binding (PPB)	95.9%	92.3%	57.1%	96.3%	84.9%
Metabolism					
CYP1A2 inhibitor	−−−	−−−	−−−	−−−	+++
CYP1A2 substrate	-	−−−	++	+++	-
CYP2C19 inhibitor	--	−−−	−−−	--	+
CYP2C19 substrate	+++	−−−	+++	+++	-
CYP2C9 inhibitor	−−−	−−−	−−−	−−−	--
CYP2C9 substrate	−−−	−−−	+	+++	++
CYP2D6 inhibitor	++	−−−	−−−	−−−	−−−
CYP2D6 substrate	+++	−−−	−−−	+++	+++
CYP3A4 inhibitor	--	--	−−−	-	−−−
CYP3A4 substrate	+++	--	++	--	+
HLM stability	+++	+++	-	+++	-
Excretion					
CLplasma	8.783	7.229	10.331	10.649	6.569
T1/2	1.237	1.211	1.668	1.031	0.75
Toxicity					
hERG blockers	0.25	0.153	0.051	0.056	0.072
Carcinogenicity	0.403	0.771	0.85	0.719	0.898
Human hepatotoxicity	0.595	0.741	0.658	0.765	0.486
AMES toxicity	0.379	0.84	0.487	0.221	0.669
Drug likeness rules					
Lipinski rule	Yes	Yes	Yes	Yes	Yes
Pfizer rule	Yes	Yes	Yes	No	Yes
GSK rule	Yes	No	Yes	No	Yes
Golden triangle	Yes	Yes	Yes	Yes	Yes
Medicinal chemistry					
PAINS	0 alert	0 alert	0 alert	0 alert	0 alert
Brenk	0 alert	2 alerts	0 alert	1 alert	1 alert
Leadlikeness	3 violations: MW > 350, Rotors>7, XLOGP3>3.5	1 violation: MW > 350	Yes	1 violation: MW < 250	1 violation: MW < 250
Synthetic accessibility	3.91	5.93	4.22	4.17	3.01

BBB, Blood-Brain Barrier; HIA, human intestinal absorption; CLplasma, Plasma Clearance; T1/2, Half-Life. For classification endpoints, prediction probability values are represented by the following symbols: 0–0.1 (−−−), 0.1–0.3 (−−), 0.3–0.5 (−), 0.5–0.7 (+), 0.7–0.9 (++), and 0.9–1.0 (+++).

Metabolic profiling indicated compound-specific cytochrome P450 interactions. Nardostachysin showed minimal CYP interactions across all isoforms, suggesting reduced drug-drug interaction risks, while oroselol emerged as a CYP1A2 inhibitor (+++) and virolin as a CYP2D6 inhibitor (++). Human liver microsomal stability was high (+++) for virolin, nardostachysin, and bulnesol.

Excretion parameters revealed short half-lives (0.75–1.668 h) and moderate plasma clearance (6.569–10.649 mL/min/kg). The BOILED-Egg analysis (Figure 17) confirmed gastrointestinal absorption potential for all compounds, with only bulnesol showing additional BBB penetration capability.

In silico-based toxicity assessment revealed variable safety profiles (Table 5). hERG blocking potential was lowest for nardosinonediol (0.051) and highest for virolin (0.25). Carcinogenicity predictions ranged from 0.403 (virolin) to 0.898 (oroselol), while human hepatotoxicity potential was lowest for oroselol (0.486). Acute toxicity profiling (Table 6) indicated nardostachysin had superior safety with negative predictions across all endpoints, while virolin showed positive predictions for inhalation toxicity and skin sensitization.

**TABLE 6:** Acute toxicity predictions across exposure routes of selective Bioactives analyzed by StopTox.

Compound	Acute inhalation toxicity	Acute oral toxicity	Acute dermal toxicity	Eye irritation and corrosion	Skin sensitization	Skin irritation and corrosion
Virolin	Toxic (+)	Non-toxic (−)	Non-toxic (−)	Non-toxic (−)	Sensitizer (+)	Negative (−)
Nardostachysin	Non-toxic (−)	Non-toxic (−)	Non-toxic (−)	Non-toxic (−)	Non-sensitizer (−)	Negative (−)

Drug-likeness scores were 0.33 for virolin and 0.01 for nardostachysin (Figure 18), with ProTox 3.0 classification placing them in Toxicity Classes 4 and 2, respectively. Medicinal chemistry compliance showed all compounds passed PAINS filters, confirming absence of promiscuous binding motifs, though variable Brenk alerts and lead-likeness violations were observed (Table 5).

## 4 Discussion

*N. jatamansi* methanolic extract exhibited superior dual enzyme inhibitory activity surpassing the clinical standard acarbose against both α-glucosidase and α-amylase, with preferential α-glucosidase selectivity and distinctive sigmoidal dose-response kinetics contrasting with acarbose’s linear behavior. This remarkable bioactivity pattern and cooperative binding characteristics suggest orchestrated contributions from synergistic interactions among structurally diverse bioactive phytochemicals within the complex sesquiterpenoid-rich matrix (Chaachouay and Zidane, 2024). Understanding which specific molecular constituents drive this enhanced therapeutic potential required integrated computational-experimental investigation combining literature-based virtual screening with advanced molecular modeling approaches to identify lead compounds and elucidate binding mechanisms (Agu et al., 2023).

Published phytochemical investigations establish that *N*. *jatamansi* rhizomes contain predominantly sesquiterpenoids, with jatamansone, nardostachone, and aristolone derivatives collectively comprising 60%–75% of extractable compounds (Bian et al., 2021; Sahu et al., 2016). Nardosinone type sesquiterpenes have been quantified at ∼2%–3% w/w in certain preparations (Bian et al., 2021). Regarding our lead compounds nardostachysin was first characterized structurally as a terpenoid ester with fused guaiane skeleton (Chatterjee et al., 2000), occurring at approximately 0.1%–0.3% w/w based on reported isolation yields, though precise quantitative data remain limited. Virolin appears in trace amounts in essential oil fractions (Ahn et al., 2001). Critically, current study lacks phytochemical profiling of our specific extract; therefore, virolin and nardostachysin concentrations in the tested material cannot be confirmed. This limitation necessitates future LC-MS/MS profiling coupled with isolation and NMR characterization to correlate computational predictions with actual constituent levels.

Our selection prioritized computational binding affinity over abundance, a validated strategy in natural product drug discovery where minor constituents frequently demonstrate superior target specificity (Atanasov et al., 2021). This approach is justified by published evidence: nardosinone type sesquiterpenes suppress NF-κB and MAPK signalling, reducing inflammatory mediators (iNOS, COX-2, TNF-α, IL-1β) (Yoon et al., 2018a; Ko et al., 2018), while Nardostachysin containing extracts enhance serotonin transporter activity with antidepressant effects (Li et al., 2021) Virolin exhibits LDL antioxidant activity (IC_50_ = 4.3 μM), relevant to diabetic vascular complications (Ahn et al., 2001). This multi-target pharmacology enzyme inhibition plus antioxidant/anti-inflammatory properties positions these compounds as addressing both glucose dysregulation and diabetic complications.

Our docking analysis revealed distinct binding architectures compared to acarbose. While acarbose functions as substrate mimetic through extensive hydrogen bonding characteristic of pseudotetrasaccharides (Borges de Melo et al., 2006), virolin achieved −9.6 kcal/mol against α-glucosidase (1.0 kcal/mol superior to acarbose’s −8.6 kcal/mol) through tripartite engagement: catalytic residues (Asp199, Glu256, Arg197), substrate recognition elements (Tyr63, Phe144), and hydrophobic regions (Leu285, Phe282). This multi-site network three hydrogen bonds, dual π-π stacking with Phe282, π-anion interaction with Asp326 represents structurally distinct inhibition via aromatic-dominated interactions rather than solely polar contacts.

Nardostachysin demonstrated −9.5 kcal/mol against α-amylase, a remarkable 2.4 kcal/mol advantage over acarbose (−7.1 kcal/mol). Dual hydrogen bonding with catalytic triad members Asp300 and His305, combined with π-sigma and π-alkyl interactions with Trp59, created comprehensive catalytic site occupancy contrasting with acarbose’s 11 peripheral bonds lacking equivalent catalytic precision (Agu et al., 2023).

Contextualization within sesquiterpene studies reveals instructive patterns. Investigation of *Persicaria hydropiper* essential oils identified β-caryophyllene epoxide as the most potent sesquiterpene (−8.3050 kcal/mol α-amylase; −8.3182 kcal/mol α-glucosidase), with epoxide oxygen enabling strong hydrogen acceptor interactions (Mahnashi et al., 2022). Other sesquiterpenes showed distinct selectivities: nerolidol preferred α-glucosidase (−8.2988 vs. −7.0590 kcal/mol) through H-pi stacking with Phe177/Phe300, while beta element demonstrated strong α-glucosidase preference (−7.7074 vs. −6.0798 kcal/mol). A binding energy hierarchy emerged across compound classes: flavonoid glycosides (rutin: -11.68 to −11.93 kcal/mol) > triterpenoid saponins (−9.19 to −9.39) > sterols (−8.99 to −9.11) > sesquiterpene epoxides (−8.30 to −8.32) > simple sesquiterpenes (−6.08 to −8.30) (Shivanagoudra et al., 2019; Choudhury et al., 2024). Larger polycyclic structures achieve superior binding through extensive hydrogen networks, while sesquiterpenes compensate smaller size through strategic hydrophobic optimization positioning our compounds (Virolin: −9.6; Nardostachysin: −9.5 kcal/mol) among the most potent sesquiterpene based modulators reported.

The architectural differences between enzyme binding pockets rationalize selectivities. α-Amylase’s narrower catalytic cleft with concentrated negative charge (Asp197, Glu233, Asp300) favors compact structures, while α-glucosidase’s extended pocket with aromatic residues (Phe177, Phe300) and arginine contacts (Arg312, Arg439) accommodates larger molecules explaining why flavonoids achieve −11.93 kcal/mol against α-glucosidase but sesquiterpenes show balanced dual activity.

While α-glucosidase PDB ID:2ZE0 used in our computational studies is of bacterial origin, its conserved catalytic residues and extensive precedent in α-glucosidase inhibitor studies justify its use for initial screening. The GH13 family’s conserved Asp–Glu–Asp catalytic triad across species enables the effective translation of binding predictions from microbial homologs. Future comparative docking using human MGAM subunits (2QMJ, 3TOP) will further refine the translational accuracy of these findings (Rangel-Galván et al., 2024).

The 100 ns molecular dynamics simulations revealed divergent temporal behaviors. Nardostachysin-α-amylase achieved solid stability (protein RMSD: 1.8 Å; ligand RMSD <1.0 Å; ΔG = −158.51 kcal/mol) with persistent His305, Asp197, and Tyr62 contacts combining polar and hydrophobic interactions. Conversely, virolin-α-glucosidase exhibited higher fluctuations (protein RMSD: 2.5 Å) and reduced binding energy (ΔG = −82.28 kcal/mol) a 76 kcal/mol difference manifesting in late-stage dissociation (C50-Tyr267 distance expanding from 2.5–6.0 Å to 8–10 Å after 75 ns). This demonstrates that static docking scores may overestimate binding without considering dynamic effects (Zhou et al., 2025).

DFT calculations at B3LYP/6–311++G(d,p) level revealed that virolin’s HOMO-LUMO gap (4.78 eV) versus nardostachysin’s (4.57 eV) indicates marginally enhanced reactivity for the latter. Reactivity descriptors showed virolin’s higher chemical hardness (η = 2.39 eV) compared to nardostachysin (η = 2.29 eV), with electrophilicity indices (ω = 2.51 vs. 2.67 eV) suggesting nardostachysin’s superior electron-accepting capacity. However, the critical finding was the dipole moment difference: nardostachysin’s 6.29 Debye versus virolin’s 4.83 Debye a 30% polarity enhancement directly correlating with dual enzyme activity.

α-Amylase’s catalytic cleft concentrates negative charge through Asp197, Glu233, and Asp300 carboxylates (combined −3 charge) (Fisher et al., 2006). Nardostachysin’s elevated dipole (6.29 D) achieves optimal electrostatic complementarity: guaiane hydroxyl groups (partial positive on H atoms) align with anionic sites, while ester carbonyl oxygen (partial negative) engages His305’s imidazole nitrogen (partial positive). α-Glucosidase presents distributed charge with negative patches (Asp349, Asp408, Glu276) balanced by positive regions (Arg312, Arg439). Virolin’s moderate dipole (4.83 D) optimally complements this landscape: phenolic hydroxyl engages Asp199, aromatic rings participate in π-π stacking with Phe282/Tyr63, and oxygen forms π-anion contact with Asp326 while establishing strong hydrogen acceptor interactions with Arg312 (2.83 Å with −3.7 kcal/mol; 2.98 Å with −2.8 kcal/mol). Nardostachysin’s higher polarity, advantageous in α-amylase’s concentrated anionic environment, offers less benefit in α-glucosidase’s charge-distributed pocket, explaining virolin’s α-glucosidase selectivity (−9.6 vs. −9.0 kcal/mol) while nardostachysin excels against α-amylase (−9.5 vs. −7.1 kcal/mol).

DFT studies on sesquiterpene lactones from *Hertia cheirifolia* corroborate these principles: HOMO-LUMO gaps (4.2–5.1 eV) and dipole moments (3.8–6.7 D) correlating with α-amylase inhibition, where higher polarity enhanced catalytic engagement (Zhang et al., 2016). Frontier orbital distributions showed HOMO localization on oxygen functionalities (hydrogen bonding sites) and LUMO delocalization across aromatic systems (π-π stacking capacity) consistent with our calculated patterns.

In silico ADMET profiling revealed perfect Lipinski Rule-of-Five compliance with zero PAINS alerts for all sesquiterpenoids, validating genuine bioactivity, though experimental validation through *in vitro* (Caco-2, hepatocyte stability, CYP450 profiling) and *in vivo* pharmacokinetic studies remains essential. Critical translational barriers emerged: uniformly poor predicted oral bioavailability (HIA: −−−), high plasma protein binding (92%–96%), and short elimination half-lives (0.75–1.67 h). Metabolic profiles critically differentiated lead compounds: nardostachysin exhibited minimal CYP450 interactions with high hepatic stability (+++), representing lowest drug-drug interaction risk for diabetic polypharmacy, whereas virolin showed extensive CYP substrate activity (CYP2C19/2D6/3A4: +++) raising metabolic interaction concerns. Acute toxicity classifications revealed nardostachysin’s Class 2 (5–50 mg/kg, requiring narrow therapeutic monitoring) versus virolin’s Class 4 (300–2,000 mg/kg, supporting development feasibility) with inhalation/skin sensitization constraining formulation options.

Comparative analysis with acarbose provides instructive context: acarbose’s <2% oral bioavailability enables local intestinal action with minimal systemic exposure, undergoes bacterial rather than CYP450 metabolism, but causes 50%–70% gastrointestinal adverse events suggesting sesquiterpenes’ predicted low bioavailability may paradoxically offer therapeutic advantages through site-specific action, requiring *in vivo* validation. Published ADMET studies on structurally related sesquiterpenes validate formulation strategies: essential oil preparations bypass first-pass metabolism (Cornara et al., 2020), nano-encapsulation and cyclodextrin complexation achieve 30%–40% bioavailability improvements (Jaradat et al., 2020), and self-emulsifying drug delivery systems (SEDDS) exploit favorable lipophilicity (LogP 2.11–3.41), transforming poor absorption into controlled-release opportunities for clinical translation.

## 5 Conclusion

Experimental validation confirmed *N. jatamansi* extract’s 2-fold superior enzyme inhibition over acarbose, while computational screening identified Virolin and Nardostachysin as promising lead candidates with distinct binding mechanisms. Molecular dynamics simulations are critically distinguished between static binding predictions and temporal stability, revealing Nardostachysin’s sustained complex formation as the more viable therapeutic candidate. Quantum mechanical analyses elucidated the electronic determinants of enzyme selectivity through calculated dipole moments and frontier orbital energies. However, these remain virtual screening predictions requiring experimental validation, as the compounds were not isolated or individually tested. Future studies must prioritize compound isolation from *N. jatamansi*, *in vitro* enzyme inhibition validation, *in vivo* pharmacological efficacy in diabetic models, and rational formulation strategies to translate these computational predictions into clinically viable antidiabetic therapeutics.

## Data Availability

The original contributions presented in the study are included in the article/Supplementary Material, further inquiries can be directed to the corresponding author.
