# Supplementary material for: Computational-experimental integration identifies potent carbohydrate-hydrolyzing enzyme inhibitors from Nardostachys jatamansi: molecular docking, dynamics and pharmacokinetic predictions
Source: Front Pharmacol. 2026 Jan 12;16:1713452. doi: 10.3389/fphar.2025.1713452 (PMC12833380; doi:10.3389/fphar.2025.1713452)
Supplement: Supplementary file 1 [file Table1.docx]

***Supplementary Material***

Supplementary table S1: Phytochemicals of *Nardostachys jatamansi* with Classifications

| **S.No.** |  | **Compound Name** | **Chemical Class** |
| --- | --- | --- | --- |
| 1 | 10726905 | (-)-7-Epi-alpha-selinene | Sesquiterpene |
| 2 | 10198387 | (-)-Valeranone | Monoterpene ketone |
| 3 | 90805 | (+)-gamma-Gurjunene | Sesquiterpene |
| 4 | 5320089 | (1aR,7bS)-1,1,7,7a-tetramethyl-2,4,5,6,7,7b-hexahydro-1aH-cyclopropa[a]naphthalen-4-ol | Sesquiterpene alcohol |
| 5 | 15560278 | (1aR,7R,7aR,7bS)-1,1,7,7a-tetramethyl-2,3,5,6,7,7b-hexahydro-1aH-cyclopropa[a]naphthalene | Sesquiterpene |
| 6 | 6324867 | (1aR,7S,7bS)-1,1,7,7a-tetramethyl-1a,2,3,5,6,7b-hexahydrocyclopropa[a]naphthalen-7-ol | Sesquiterpene alcohol |
| 7 |  | (1S,5S,9R)-9-Isopropyl-1-methyl-6-methylenespiro [4.5] decan-1-ol | Sesquiterpene alcohol |
| 8 |  | (1S,6S,7S,10S)-4,10-dimethyl-7-prop-1-en-2-yltricyclo [4.4.0.01,5] decane | Sesquiterpene |
| 9 | 10466564 | (4R,4aR,5R)-4-(2-hydroxypropan-2-yl)-4a,5-dimethyl-4,5,6,7-tetrahydronaphthalen-1-one | Sesquiterpene ketone |
| 10 | 91723653 | (S,1Z,6Z)-8-Isopropyl-1-methyl-5-methylenecyclodeca-1,6-diene | Sesquiterpene |
| 11 | 11002035 | (S)-8,8-Dimethyl-2-oxo-2,8,9,10-tetrahydropyrano[2,3-f] chromen-9-yl 2-methylbutanoate | Coumarin ester |
| 12 | 68171 | 1-Hexacosanol | Fatty alcohol |
| 13 | 68406 | 1-Octacosanol | Fatty alcohol |
| 14 | 71717616 | 1(10)-Aristolen-2-one | Sesquiterpene ketone |
| 15 | 26049 | 3-Carene | Monoterpene |
| 16 | 72 | 3,4-Dihydroxybenzoic acid | Phenolic acid |
| 17 | 11230 | 4-Carvomenthenol | Monoterpene alcohol |
| 18 | 520743 | 7-Hexadecene | Alkene |
| 19 | 134856755 | 8-(2-Hydroxypropan-2-yl) furo[2,3-h] chromen-2-one | Furanocoumarin |
| 20 | 6407889 | 8,8-dimethyl-2H,8H-pyrano[2,3-f] chromene-2,9(10H)-dione | Coumarin |
| 21 | 68231 | Actinidine | Alkaloid |
| 22 | 6419725 | alpha-Carotene | Carotenoid |
| 23 | 70678558 | alpha-Copaene | Sesquiterpene |
| 24 | 92139 | alpha-Curcumene | Sesquiterpene |
| 25 | 15560276 | alpha-Gurjunene | Sesquiterpene |
| 26 | 521710 | alpha-Patchoulene | Sesquiterpene |
| 27 | 6654 | alpha-Pinene | Monoterpene |
| 28 | 10856614 | alpha-Selinene | Sesquiterpene |
| 29 | 17100 | alpha-Terpineol | Monoterpene alcohol |
| 30 | 643915 | Angelic acid | Organic acid |
| 31 | 10658 | Angelicin | Furanocoumarin |
| 32 | 530421 | Aristolene | Sesquiterpene |
| 33 | 10104370 | beta-Bisabolene | Sesquiterpene |
| 34 | 5281515 | beta-Caryophyllene | Sesquiterpene |
| 35 | 57339298 | beta-Copaene | Sesquiterpene |
| 36 | 6918391 | beta-Elemene | Sesquiterpene |
| 37 | 91457 | beta-Eudesmol | Sesquiterpene alcohol |
| 38 | 15560252 | beta-Guaiene | Sesquiterpene |
| 39 | 6450812 | beta-Gurjunene | Sesquiterpene |
| 40 | 638014 | beta-Ionone | Monoterpene ketone |
| 41 | 101596917 | beta-Maaliene | Sesquiterpene |
| 42 | 101731 | beta-Patchoulene | Sesquiterpene |
| 43 | 14896 | beta-Pinene | Monoterpene |
| 44 | 442393 | beta-Selinene | Sesquiterpene |
| 45 | 222284 | beta-Sitosterol | Sterol |
| 46 | 93009 | Bornyl acetate | Monoterpene ester |
| 47 | 12302134 | Bulnesol | Sesquiterpene alcohol |
| 48 | 6429077 | Calamenene | Sesquiterpene |
| 49 | 442347 | Carotol | Sesquiterpene alcohol |
| 50 | 1742210 | Caryophyllene oxide | Sesquiterpene oxide |
| 51 | 442359 | Caswell No. 264AB | Synthetic preservative |
| 52 | 11276107 | Cubebol | Sesquiterpene alcohol |
| 53 | 442363 | Daucol | Sesquiterpene alcohol |
| 54 | 94275 | delta-Guaiene | Sesquiterpene |
| 55 | 8215 | Docosanoic acid | Fatty acid |
| 56 | 92138 | Elemol | Sesquiterpene alcohol |
| 57 | 2758 | Eucalyptol | Monoterpene oxide |
| 58 | 445858 | Ferulic acid | Phenolic acid |
| 59 | 521302 | gamma-Patchoulene | Sesquiterpene |
| 60 | 11467 | gamma-Terpineol | Monoterpene alcohol |
| 61 | 5281519 | Germacrene B | Sesquiterpene |
| 62 | 12407 | Hexacosane | Alkane |
| 63 | 11006 | Hexadecane | Alkane |
| 64 | 5281520 | Humulene | Sesquiterpene |
| 65 | 275468748 | Icosanoic acid hexacosyl ester | Fatty acid ester |
| 66 | 10430 | Isovaleric acid | Branched fatty acid |
| 67 |  | Jatamansic acid | Sesquiterpene acid |
| 68 | 668081 | Jatamansin | Coumarin |
| 69 | 6429183 | Jatamol A | Sesquiterpene alcohol |
| 70 | 274137028 | Jatamol B | Sesquiterpene alcohol |
| 71 | 11074994 | Ledol | Sesquiterpene alcohol |
| 72 | 5317025 | Linarin | Flavone glycoside |
| 73 | 600670 | Lomatin | Coumarin |
| 74 | 259846 | Lupeol | Triterpene |
| 75 | 31253 | Myrcene | Monoterpene |
| 76 | 10582 | Myrtenol | Monoterpene alcohol |
| 77 | 5318034 | n-Hexacosanyl isovalerate | Fatty acid ester |
| 78 |  | Nardol | Sesquiterpene alcohol |
| 79 | 168136 | Nardosinone | Sesquiterpene ketone |
| 80 | 134715257 | Nardosinonediol | Sesquiterpene diol |
| 81 |  | Nardostachone | Sesquiterpene ketone |
| 82 | 10598736 | Nardostachysin | Sesquiterpene derivative |
| 83 | 8141 | Nonane | Alkane |
| 84 | 609798 | Norseychellanon | Sesquiterpene ketone |
| 85 | 10494 | Oleanolic acid | Triterpene |
| 86 | 74477 | Oroselone | Coumarin |
| 87 | 7463 | p-Cymene | Monoterpene |
| 88 | 10955174 | Patchouli alcohol | Sesquiterpene alcohol |
| 89 | 73399 | Pinoresinol | Lignan |
| 90 | 5320651 | Pogostol | Sesquiterpene alcohol |
| 91 | 1032 | Propionic acid | Organic acid |
| 92 | 68229 | Seselin | Coumarin |
| 93 | 22211634 | Seychellene | Sesquiterpene |
| 94 | 92785 | Taraxerone | Triterpene |
| 95 | 6429302 | trans-alpha-Bergamotene | Sesquiterpene |
| 96 | 90473619 | Tricyclo(6.3.1.02,5) dodecan-1-ol, 4,4,8-trimethyl-, (1R,2S,5R,8S)- | Sesquiterpene alcohol |
| 97 | 64945 | Ursolic acid | Triterpene |
| 98 | 9855795 | Valencene | Sesquiterpene |
| 99 | 171455 | Valeranone | Monoterpene ketone |
| 100 | 6440407 | Virolin | Coumarin |
| 101 | 5284507 | Nerolidol | Sesquiterpene alcohol |
| 102 |  | 1-[2-Methyl-2-(4-Methyl-3-Pentenyl) Cyclopropyl] Ethanol | Cyclopropyl alcohol |
| 103 | 11996452 | Viridiflorol | Sesquiterpene alcohol |
| 104 | 6430906 | 6-(p-Tolyl)-2-methyl-2-heptenol | Aromatic alcohol |
| 105 |  | 2-Naphthalenemethanol | Naphthalene derivative |
| 106 | 11053257 | Spirojatamol | Sesquiterpene alcohol |
| 107 |  | Selina-6-En-4-Alpha-Ol | Sesquiterpene alcohol |
| 108 | 10398656 | Alpha-Cadinol | Sesquiterpene alcohol |
| 109 | 73555482 | Bergamotol | Sesquiterpene alcohol |
| 110 |  | 1,4-Dimethyl-7-(Prop-1-En-2-Yl) Decahydroazulen-4-Ol | Sesquiterpene alcohol |
| 111 |  | 3-Cyclohexen-1-Ol | Sesquiterpene alcohol |
| 112 | 520758 | Shyobunol | Sesquiterpene alcohol |
| 113 |  | 1-Heptatriacotanol | Long-chain fatty alcohol |
| 114 |  | Isolongifolen, 9,10-Dehydro | Sesquiterpene |
| 115 | 12303906 | Ylangenal | Sesquiterpene aldehyde |
| 116 |  | 1H-Cycloprop[E]Azulen-7-Ol | Sesquiterpene alcohol |
| 117 | 91746597 | 1,1,4,7-Tetramethyldecahydro-1H-cyclopropa[e]azulen-4-ol | Sesquiterpene alcohol |
| 118 | 6440940 | Valerenic Acid, Methyl Ester | Fatty acid ester |
| 119 |  | Hexadecanoic Acid, Methyl Ester | Fatty acid ester |
| 120 | 273568 | 7-Isopropenyl-1,4a-Dimethyl-4,4a,5,6,7,8-Hexahydro-3H-Naphthalen-2-One | Sesquiterpene ketone |
| 121 | 6427490 | Cedren-13-Ol | Sesquiterpene alcohol |
| 122 |  | Ethyl Oleate | Fatty acid ester |
| 123 |  | Hexadecenoic Acid, Ethyl Ester | Fatty acid ester |
| 124 | 6536796 | Lanceol | Sesquiterpene alcohol |
| 125 | 91723244 | Aristol-1[10]-En-9-Yl Isovalerate | Sesquiterpene ester |
| 126 | 91730081 | (E)-Valerenyl isovalerate | Sesquiterpene ester |
| 127 | 5284421 | 9,12-Octadecadienoic Acid (Z,Z)-, Methyl Ester | Fatty acid ester |
| 128 |  | 6-Octadecenoic Acid, Methyl Ester | Fatty acid ester |
| 129 | 14587730 | Gerany-p-cymene | Monoterpene derivative |
| 130 | 5282184 | Linoleic acid ethyl ester | Fatty acid ester |
| 131 |  | Heptadecanoic Acid, Ethyl Ester | Fatty acid ester |
| 132 | 102115341 | Humulenol-II | Sesquiterpene alcohol |
| 133 | 457801 | Gamma-Sitosterol | Sterol |
| 134 | 579897 | Gamma-Sitostenone | Sterol derivative |
| 135 | 10198387 | Jatamansone | Sesquiterpenoid |
| 136 | 28481 | Calarene | Sesquiterpenoid |
| 137 | 759302 | Jatamansinol | Coumarin |
| 138 | 160600 | Oroselol | Coumarin |
| 139 | 9859337 | Valerianol | Octahydronaphthalenes |
| 140 | 4229818 | Nardin | Sesquiterpenes |
| 141 | 134586 | Pyranocoumarin | Coumarins |
| 142 | 668081 | Xanthogalin | Alkaloid |
| 143 | 71583464 | Nardoaristolones B | Nor-sesquiterpenoid |
| 144 | 6429378 | Valeranol | Octahydronaphthalenes |
